# Inactivation of Tumor Suppressor CYLD Inhibits Fibroblast Reprogramming to Pluripotency

**DOI:** 10.3390/cancers15204997

**Published:** 2023-10-15

**Authors:** Nikolaos Bekas, Martina Samiotaki, Maria Papathanasiou, Panagiotis Mokos, Athanasios Pseftogas, Konstantinos Xanthopoulos, Dimitris Thanos, George Mosialos, Dimitra Dafou

**Affiliations:** 1 School of Biology, Aristotle University of Thessaloniki, 54124 Thessaloniki, Greece; mpekasns@bio.auth.gr (N.B.); panagiotis.mokos@gmail.com (P.M.); gmosialo@bio.auth.gr (G.M.); 2 Biomedical Sciences Research Center “Alexander Fleming”, 16672 Vari, Greece; samiotaki@fleming.gr; 3 Biomedical Research Foundation Academy of Athens, 11527 Athens, Greece; mpapathan86@gmail.com (M.P.); thanos@bioacademy.gr (D.T.); 4 Division of Experimental Oncology, IRCCS San Raffaele Hospital, Vita-Salute San Raffaele University, 20132 Milan, Italy; pseftogkas.athanasio@hsr.it; 5 Laboratory of Pharmacology, Department of Pharmacy, School of Health Sciences, Aristotle University of Thessaloniki, 54124 Thessaloniki, Greece; xantho@pharm.auth.gr

**Keywords:** CYLD, somatic cell reprogramming, MET, EMT, ECM organization, TGF-beta, pluripotency, dedifferentiation

## Abstract

**Simple Summary:**

Many aspects of the regulatory mechanisms of somatic cell reprogramming—the conversion of any cell type into pluripotent stem cells—still remain elusive. The tumor suppressor CYLD regulates several signaling pathways involved in this process. However, its potential role in reprogramming has not been investigated. In this work, we present evidence that CYLD exerts important regulatory control at the early stages of reprogramming. Loss of CYLD catalytic activity leads to the reduced reprogramming efficiency of mouse embryonic fibroblasts. Whole proteome analysis during early reprogramming stages revealed that CYLD DUB deficiency impedes a vital early reprogramming step known as the mesenchymal-to-epithelial transition (MET). Our findings expand our knowledge of early reprogramming mechanics and reveal a novel role for CYLD as an extracellular matrix regulator.

**Abstract:**

*CYLD* is a tumor suppressor gene coding for a deubiquitinating enzyme that has a critical regulatory function in a variety of signaling pathways and biological processes involved in cancer development and progression, many of which are also key modulators of somatic cell reprogramming. Nevertheless, the potential role of *CYLD* in this process has not been studied. With the dual aim of investigating the involvement of *CYLD* in reprogramming and developing a better understanding of the intricate regulatory system governing this process, we reprogrammed control (*CYLD^WT/WT^*) and CYLD DUB-deficient (*CYLD^Δ9/Δ9^*) mouse embryonic fibroblasts (MEFs) into induced pluripotent stem cells (iPSCs) through ectopic overexpression of the Yamanaka factors (Oct3/4, Sox2, Klf4, c-myc). CYLD DUB deficiency led to significantly reduced reprogramming efficiency and slower early reprogramming kinetics. The introduction of WT CYLD to *CYLD^Δ9/Δ9^* MEFs rescued the phenotype. Nevertheless, CYLD DUB-deficient cells were capable of establishing induced pluripotent colonies with full spontaneous differentiation potential of the three germ layers. Whole proteome analysis (Data are available via ProteomeXchange with identifier PXD044220) revealed that the mesenchymal-to-epithelial transition (MET) during the early reprogramming stages was disrupted in *CYLD^Δ9/Δ9^* MEFs. Interestingly, differentially enriched pathways revealed that the primary processes affected by CYLD DUB deficiency were associated with the organization of the extracellular matrix and several metabolic pathways. Our findings not only establish for the first time CYLD’s significance as a regulatory component of early reprogramming but also highlight its role as an extracellular matrix regulator, which has profound implications in cancer research.

## 1. Introduction

Somatic cell reprogramming is a process by which somatic cells can be transformed into pluripotent stem cells [1]. Although many methodologies have been developed to achieve this goal, such as chemical reprogramming [2,3,4], plasmid-based [5], mRNA-based [6], direct delivery [7] and piggybac transposition [8], the most widespread and well-studied is the virus-delivered, transgenic overexpression of *OCT3/4*, *SOX2*, *KLF4* and *c-myc*, also known as Yamanaka factors, which can be applied in numerous cutting-edge fields [9], such as disease modeling [10,11,12], rejuvenation [13] and tissue regeneration [14,15,16]. Despite the significant strides made in understanding the mechanisms involved in this process, reprogramming remains a remarkably complex phenomenon, with a large number of heterogeneous factors regulating both reprogramming progression and efficiency [17,18,19,20]. Just a few examples include the implication of numerous independently acting genes [21,22,23,24,25], small molecules [26,27,28,29], most studied signaling pathways [30,31,32,33,34,35,36,37,38], epigenetic modifications [39,40,41,42,43,44] and micro-RNAs [45,46,47,48]. Understanding the involvement of each of these heterogeneous mechanisms and then combining them is imperative to fully decipher the regulatory network of reprogramming.

Several pieces of evidence indicate that somatic cell reprogramming resembles the process of carcinogenesis. The Yamanaka factors are known oncogenes implicated in various forms of cancer and also associated with poor prognosis [49,50,51,52,53,54,55,56,57,58,59]. In addition, tumor suppressor genes, such as p53 and p21, are reprogramming barriers, and loss of their activity can dramatically increase reprogramming efficiency [60,61,62]. Moreover, many changes observed during reprogramming, such as the inhibition of apoptosis and senescence, increased proliferation rates, epithelial-to-mesenchymal transition and the Warburg effect, are well-established and common processes in tumor development [20,63,64]. Furthermore, during early reprogramming, cells undergo drastic morphological changes [65], such as the loss of somatic cell identity and the establishment of new intercellular contacts [66,67], phenomena also observed in tumorigenesis [68,69]. Based on the above, it has been suggested that reprogramming can be used not only as a model for the study of numerous cancer-related processes, including the EMT [64], development and differentiation of cancer stem cells [70,71,72] and oncogenic transformation [73,74,75,76,77], but also for the discovery of novel diagnostic and therapeutic targets [72,78,79].

*CYLD* is a 60 kb long gene encoding a 956-amino-acid protein (CYLD) that functions primarily as a deubiquitinating enzyme [80]. The contribution of CYLD to the regulation of NF-κB activity has been extensively studied [80,81], where it appears to act as both a K-63 and M-1 deubiquitinase [82,83,84]. Furthermore, CYLD has been shown to have a regulatory role in other signaling pathways, including TGF-beta [85,86,87], Wnt-beta catenin [88,89], Hippo [85,90], JNK [91,92,93], ERK [94] and Akt [95,96,97]. Regarding its biological role, CYLD has been studied mainly in terms of its role in carcinogenesis. It has been shown to be a tumor suppressor gene, as both a decrease in its expression and loss of function have been associated with adverse effects in many cancers [85,87,88,91,92,93,98,99,100,101]. Despite that in most cases the molecular basis of CYLD involvement remains elusive, in some types of cancer a relevant molecular mechanism has been identified. In breast cancer and head and neck squamous cell carcinoma (HNSCC), CYLD DUB deficiency leads to TGF-beta pathway activation and the promotion of epithelial-to-mesenchymal transition (EMT) [85,87]. On the other hand, the role of CYLD in multiple myeloma depends mainly on the regulation of WNT, as the loss of CYLD leads to pathway hyperactivation and cancer aggressiveness [88]. The regulation of NF-κB by CYLD plays a critical role in lung cancer [101,102], B-cell receptor-dependent lymphomas [103], glioblastoma [104] and pulmonary adenocarcinoma [105], among others.

CYLD may also have a regulatory role in cellular reprogramming. As already mentioned, CYLD is a tumor suppressor gene and modulates several pathways crucial in reprogramming progression, most notably TGF-beta, Hippo and Wnt. Moreover, in some cases, CYLD DUB deficiency has been associated with cancer stemness [104,106,107]. In this study, we reveal for the first time the regulatory role of CYLD in somatic cell reprogramming. CYLD DUB deficiency leads to a decrease in reprogramming efficiency, without affecting the pluripotency potential of induced stem cells and the in vitro spontaneous differentiation ability. CYLD DUB-deficient cells are characterized by the slower induction of pluripotency and expression of epithelial markers and deactivation of mesenchymal genes, particularly those related to the organization of the extracellular matrix (ECM). Taken together, we propose that CYLD is a key regulator of successful early reprogramming development.

## 2. Materials and Methods

### 2.1. Generation of Lentiviral Particles for Somatic Cell Reprogramming

The reprogramming protocol used is based on the co-expression of the OSKM reprogramming cassette using the polycistronic vector TetO-FUW-OSKM (TetO-FUW-OSKM was a gift from Rudolf Jaenisch (Addgene plasmid # 20321; https://www.addgene.org/20321/, accessed on 14 October 2023)). The reprogramming cassette is doxycycline-inducible, based on the tet-on system, and its expression is controlled by the M2rtTA protein. As an M2rtTA vector, we used FUW-M2rtTA (FUW-M2rtTA was a gift from Rudolf Jaenisch (Addgene plasmid #20342; https://www.addgene.org/20342/, accessed on 14 October 2023)). Standard HBS calcium phosphate transfection was used for the production of lentiviral particles in human embryonic kidney cells 293T (HEK293T). The plasmids pMD2.G (pMD2.G was a gift from Didier Trono (Addgene plasmid # 12259; https://www.addgene.org/12259/, accessed on 14 October 2023)) and psPAX2 (psPAX2 was a gift from Didier Trono (Addgene plasmid # 12260; https://www.addgene.org/12260/, accessed on 14 October 2023)) were utilized as envelope and packaging vectors, respectively. Briefly, the culture medium was changed 4 h before transfection. The transfection cocktail consisted of 50% HBS 2X, 5% CaCl_2_ 2 M, 10 μg of packaging and envelope plasmids and 20 μg of transfer plasmids. Three days after the medium change in transfected HEK293T, lentivirus-containing supernatants were collected for further use.

### 2.2. Isolation of Mouse Embryonic Fibroblasts (MEFs)

MEFs were generated as previously described [108]. *CYLD^WT/WT^* (WT) and *CYLD^Δ9/Δ9^* (Δ9) MEFs were used for somatic cell reprogramming experiments, whereas *CYLD^WT/Δ9^* MEFs were utilized for feeder cell preparation. Initial experiments were optimized with matching genetic background feeders and *CYLD^WT/Δ9^* cells. No difference in any experimental outcome was observed, so the scaled-up experiments were performed using only heterozygote MEF feeder cells, as we could reduce the amount of animal use also. Three independent MEF cell lines were established, two of them derived from littermate embryos of the same crossing (crossing b2, cell lines b2_1 and b2_2) and the third one from littermate embryos of a different crossing (crossing b3, cell lines b3). All subsequent experiments (reprogramming efficiency/kinetics/whole proteome analysis, pluripotency maintenance, in vitro spontaneous differentiation) were conducted using the three biological replicates described above. Finally, each biological replicate was analyzed in at least three technical replicates for each experiment presented.

### 2.3. Genotyping

Genotyping of animals and cells was conducted according to the protocol described by Trompouki et al. [108] and Karatzas et al. [109]. Briefly, genomic DNA from selected samples was isolated by using the PureLink™ Genomic DNA Mini Kit (Invitrogen™, Waltham, MA, USA). The following PCR primers were used to characterize the CYLD locus: FWD1: 5 -GATGGCTCTTGTCACCACTT-3′, F6: 5′-CGTGAACAGATGTGATGAAGGC-3′ and R6: 5′-CTACCATCCCTGCTAACCAC-3′.

### 2.4. Production of Pinducer20 C601S and Pinducer21 FCYLD

Construction of the lentiviral expression vectors pINDUCER20 FLAGCYLDC601S and pINDUCER21 FLAGCYLDWT was performed using the Gateway protocol. In more detail, pENTR1A FLAGCYLDWT and pENTR1A FLAGCYLDC601S inserts flanked by the attL1 and attL2 regions (previously constructed in our lab) were transferred between the attR1 and attR2 regions of each of the two target vectors pINDUCER20 (Addgene plasmid #44012, https://www.addgene.org/44012/, accessed on 14 October 2023) and pINDUCER21 (Addgene plasmid #46948, https://www.addgene.org/46948/, accessed on 14 October 2023). This transfer was achieved by the LR recombination reaction using the LR Clonase II enzyme mixture (Invitrogen™, Waltham, MA, USA).

For the construction of pENTR1A FCYLDWT and pENTR1A FCYLD C601S, Gateway™ cloning (Invitrogen™, Waltham, MA, USA) was utilized. More specifically, the pENTR™ 1A Dual Selection Vector (Invitrogen, A10462) was cleaved with the enzymes KpnI (Fermentas, Waltham, MA, USA) and XhoI (Fermentas, Waltham, MA, USA). The enzyme reaction products were dephosphorylated using SAP (Fermentas, Waltham, MA, USA). Subsequently, a ligation reaction between FLAGCYLDWT, FLAGCYLDC601S and the empty pENTR1A vector was performed using T4 Ligase (Fermentas, Waltham, MA, USA) with a molar ratio of linear vector to linear insert of 1:1.

### 2.5. Western Blot

Cell lysis was performed using NP-40 buffer (50 mM Tris-Cl, pH 8.0, 150 mM NaCl and 1% NP-40) supplemented with a protease inhibitor cocktail (Sigma-Aldrich, St. Louis, MO, USA). Protein denaturation was achieved after diluting (1:1) the samples with 2× Laemmli Sample Buffer (Bio-Rad Laboratories, Hercules, CA, USA), followed by heating at 95 °C for 5 min. The samples were analyzed with SDS-PAGE, and proteins were electrophoretically transferred to PVDF membrane for Western blot analysis. Membranes were treated with blocking solution, consisting of 5% non-fat dry milk in PBST, for 1 h at RT. For the detection of CYLD, we used the cylindromatosis 1 antibody (E-4, sc-74434, Santa Cruz Biotechnology, Dallas, TX, USA), and for b-actin, we used the beta actin antibody (C-4, Santa-Cruz). As a secondary antibody, we used m-IgGκ BP-HRP (sc-516102, Santa Cruz Biotechnology, Dallas, TX, USA) for both CYLD and b-actin. In all cases, the antibodies were diluted in blocking solution, and the membrane was treated for 1 h at RT. Membrane-bound antibodies were detected with an enhanced chemiluminescence detection kit (Pierce, Waltham, MA, USA) using a Typhoon FLA 7000 imaging system (GE Healthcare Life Sciences, Chicago, IL, USA). The E-4 CYLD antibody interacts with the C-terminal 419 amino acids of human CYLD.

### 2.6. Cell Culture

MEFs were cultured on plates treated with a 0.1% gelatin solution (20 min on 37 °C). MEF culturing medium consisted of high-glucose DMEM, 10% FBS, 1X NEAA, 1X penicillin–streptomycin, 1X GlutaMAX^TM^ supplement (Gibco-Invitrogen, Waltham, MA, USA) and 0.1 mM β-mercaptoethanol. Induced pluripotent stem cells (iPSCs) were cultured on feeder cells plated onto gelatin-coated plates. iPSC medium consisted of high-glucose DMEM (Gibco-Invitrogen, Waltham, MA, USA), 15% KnockOut™ Serum Replacement (Gibco-Invitrogen, Waltham, MA, USA), 1X NEAA (Gibco-Invitrogen, Waltham, MA, USA), 1X penicillin–streptomycin (Gibco-Invitrogen, Waltham, MA, USA), 1X GlutaMAX^TM^ supplement (Gibco-Invitrogen, Waltham, MA, USA), 0.1 mM β-mercaptoethanol (Gibco-Invitrogen, Waltham, MA, USA) and 10 ng/mL mouse leukemia inhibitory factor (LIF Recombinant Mouse Protein, embryonic stem cell-qualified, Gibco-Invitrogen, Waltham, MA, USA). All cell types used were tested for mycoplasma.

### 2.7. Feeder Cell Preparation

*CYLD^WT/Δ9^* MEFs were treated with a mitomycin C (Sigma-Aldrich, St. Louis, MO, USA) solution (10 μg/mL in DMEM) for 2 h at 37 °C. Cells were subsequently washed with PBS and immediately trypsinized and cryopreserved for future use.

### 2.8. Somatic Cell Reprogramming

Reprogramming was conducted according to Papathanasiou et al. [110]. The day before infection, MEFs were passaged at a ratio of 5 × 10^5^ cells/p100 plate. The infection cocktail was prepared with a ratio of 3 mL OSKM virus + 3 mL M2rtTA virus + 2 mL MEF medium/p100 plate. Cells were cultured for 24 h with the infection cocktail, which was then replaced with MEF medium. The next day, a DOX-containing medium (2 μg/mL) was added to the cells, which initiated the reprogramming process (D0). On day 6, early iPSC colonies were dissociated with accutase and plated on wells with a ratio of 10^5^ cells/p100 plate and cultured with DOX-containing (2 μg/mL) iPSC medium. Cells were cultured until day 18, when reprogramming efficiency was calculated via ALP staining.

Apart from the three MEF cell lines (mentioned in Section 2.2), two additional derivative cell lines were established using the three separate Δ9 MEF isolates. For the first one, we inserted into Δ9 MEFs a plasmid expressing the WT CYLD (from now on termed Δ9 FCYLD), and for the second one, we inserted a plasmid expressing another catalytically inactive form of CYLD known as C601S (from now on termed Δ9 C601S). The difference between C601S and Δ9 is that although both are catalytically inactive, the former is a full-length protein, rendered inactive due to the presence of a single point mutation in the catalytic center of the protein, whereas the latter is a truncated CYLD variant lacking its carboxyl terminus, including the catalytic center. In both Δ9 FCYLD and Δ9 C601S lines, the expression of the CYLD transgene is doxycycline-inducible. At the start of reprogramming, every cell line used was of similar passage. Moreover, we ensured the Yamanaka reprogramming cassette was expressed in comparable levels in each sample (Figure S1).

### 2.9. ALP Staining

On day 18 of reprogramming, the medium was removed, and cells were fixed with a 4% PFA solution for 10 min at 4 °C. After washing with PBS (1X), cells were treated with the ALP (Alkaline Phosphatase) staining solution, containing 200 μL NBT (nitro-blue tetrazolium chloride)/BCIP (5-bromo-4-chloro-3′-indolyphosphate p-toluidine salt) stock solution (Sigma-Aldrich, St. Louis, MO, USA) in 10 mL of NTMT (NaCl, Tris-Cl, MgCl_2_, Tween-20) buffer in the dark for 10 min. Reprogramming efficiency was calculated as the number of ALP-stained colonies/number of cells plated onto feeders × 100. 

### 2.10. Colony Harvesting

Using a sharp, glass Pasteur pipette, an incision was made along the border of the iPSC colony in order to separate it from the surrounding cells. The colony was then collected with a Gilson PIPETMAN (Gilson, Madison, WI, USA) and placed in medium-containing collection tubes on ice. Only colonies from the same plate were pooled together and chosen based on their phenotypic characteristics. Once enough colonies were collected, the total sample was flash frozen and stored for future use (−80 °C). A representative example can be found in Figure S2.

### 2.11. RNA Extraction, cDNA Synthesis and Quantitative Real-Time PCR (qPCR)

RNA extraction was conducted using the NucleoSpin RNA Plus RNA purification kit (Macherey-Nagel, Düren, Germany). RNA samples were stored at −80 °C, and RNA integrity was tested with bleach agarose gel [111]. cDNA was synthesized using the PrimeScript™ 1st strand cDNA Synthesis Kit (Takara Bio USA, San Jose, CA, USA). Real-time quantitative PCRs (qPCRs) were performed in duplicates using 5 ng of cDNA per reaction amplified by SYBR FAST qPCR Master Mix (KK4602, Kapa Biosystems, Wilmington, MA, USA) in a StepOne™ Real-Time PCR System (Applied Biosystems™, Warrington, UK) and a 7500 Fast Dx Real-Time PCR Instrument (Applied Biosystems™, Warrington, UK). CYLD qPCR primers recognize a region from exon 2 to exon 3 (spanning from 645 to 753, transcript NM_173369.3). The complete list of primer sequences can be found in Table S1.

### 2.12. Immunofluorescence

Cells were fixed with a 4% PFA solution for 10 min at room temperature. Samples were subsequently treated with a permeabilization solution consisting of 0.25% PBS/Triton-X 100 for 10 min and a blocking solution consisting of 1% BSA in PBST (PBS + 0.1% Tween 20, Sigma-Aldrich, St. Louis, MO, USA) for 30 min to block unspecific binding of the antibodies. Samples were incubated with the primary antibody in blocking solution for 1 h at RT and the secondary antibody in blocking solution for 1 h at RT in the dark. Finally, cells were incubated with DAPI solution (1 μg/mL) for 1 min and visualized using a Zeiss LSM 780 confocal microscope (Zeiss, Oberkochen, Germany). The following primary antibodies were used: OCT3/4 (H-134, sc-9081, Santa Cruz Biotechnology, Dallas, TX, USA), NANOG (C-4, sc-376915, Santa Cruz Biotechnology, Dallas, TX, USA) and LIN28 (AF3757, R&D Systems, Minneapolis, MN, USA). As a secondary antibody, Alexa Fluor™ 555 Highly Cross-Adsorbed Secondary Antibody (Invitrogen™, Waltham, MA, USA) was used. More specifically, Donkey anti-Rabbit for OCT3/4, Donkey anti-Mouse for NANOG and Donkey anti-Goat for LIN28.

### 2.13. In Vitro Spontaneous Differentiation Assay

The differentiation potential of the produced WT and Δ9 iPSC cell lines was assessed via the formation of Embryoid Bodies (EBs) and their in vitro spontaneous differentiation, as described by Liao et al. [112]. Briefly, iPSCs were dissociated with accutase and cultured into low-adhesion tissue culture plates with LIF-free iPSC medium. Embryoid Bodies (EBs) were formed after 3 days of suspension culture conditions. EBs were then trypsinized, and a single-cell suspension was plated in gelatin-coated wells and cultured in MEF medium for 14 days. Samples were harvested on days 0, 1, 2, 3, 4, 7, 10 and 14 of the differentiation procedure.

### 2.14. Proteomics

Samples harvested as described in Section 2.10 were lysed with FASP buffer (0.1 M Tris-HCl pH 7.6, 4% SDS, 0.1 M DTT). Supernatants were collected and stored at −20 °C. The protein extracts were processed by tryptic digestion using the Sp3 protocol, including an alkylation step in 100 mM iodoacetamide (Acros Organics/Thermo Scientific Chemicals, Waltham, MA, USA). Then, 20 μg of beads (1:1 mixture of hydrophilic and hydrophobic SeraMag carboxylate-modified beads, Cytiva Life Sciences, Marlborough, MA, USA) was added to each sample in 50% ethanol. Protein clean-up was performed on a magnetic rack. The beads were washed two times with 80% ethanol and once with 100% acetonitrile (Fisher Scientific, Waltham, MA, USA). The proteins captured on beads were digested overnight at 37 °C under vigorous shaking (1200 rpm, Eppendorf Thermomixer, Eppendorf, Hamburg, Germany) with 1 μg Trypsin/LysC (MS grade, Promega) prepared in 25 mM ammonium bicarbonate. The next day, the peptides were purified using a modified Sp3 clean-up protocol, solubilized in the mobile phase A (0.1% Formic acid in water) and sonicated. The peptide concentration was determined through an absorbance at 280 nm measurement using NanoPhotometer^®^ P330 (Implen, Westlake Village, CA, USA). 

Samples were analyzed with a liquid chromatography–tandem mass spectrometry (LC-MS/MS) setup consisting of a Dionex Ultimate 3000 nanoRSLC (Thermo-Fisher Scientific, Waltham, MA, USA), coupled in line with a Thermo Q Exactive HF-X Orbitrap mass spectrometer (Thermo-Fisher Scientific, Waltham, MA, USA). Peptidic samples were directly injected and separated on a 25 cm long analytical C18 column (PepSep, 1.9 μm 3 beads, 75 µm ID) using a one-hour-long run, starting with a gradient of 7% Buffer B (0.1% Formic acid in 80% acetonitrile) to 35% for 40 min and followed by an increase to 45% in 5 min and a second increase to 99% in 0.5 min and then kept constant for equilibration for 14.5 min. A full MS was acquired in profile mode using a Q Exactive HF-X Hybrid Quadrupole-Orbitrap mass spectrometer, operating in the scan range of 375–1400 *m*/*z* using 120 K resolving power with an AGC of 3 × 10^6^ and maximum IT of 60 ms followed by a data-independent acquisition method using 8 Th windows (a total of 39 loop counts), each with 15 K resolving power with an AGC of 3 × 105 and max IT of 22 ms and normalized collision energy (NCE) of 26.

Orbitrap raw data were analyzed in DIA-NN (Data-Independent Acquisition by Neural Networks) [113] against the Mouse Proteome (downloaded from Uniprot, 21,946 proteins entries, downloaded 11/2022) using the library free mode of the software (version 1.8.1) allowing up to two tryptic missed cleavages and a maximum of three variable modifications/peptide. A spectral library was created from the DIA runs and used to reanalyze them (double search mode). A DIA-NN search was used with oxidation of methionine residues, N-terminal methionine excision and acetylation of the protein N-termini set as variable modifications and carbamidomethylation of cysteine residues as a fixed modification. The match between runs feature was used for all analyses, and the output (precursor) was filtered at 0.01 FDR. The protein inference was performed on the level of genes using only proteotypic peptides.

The proteomics data were processed with the Perseus software (version 1.6.15.0, Max Planck Institute of Biochemistry) [114]. Values were log(2) transformed, a threshold of 70% of valid values in at least one group was applied, and the missing values were replaced from normal distribution. For statistical analysis, a Student’s t-test was performed, and permutation-based FDR was calculated. The mass spectrometry proteomics data have been deposited to the ProteomeXchange Consortium via the PRIDE [115] partner repository with the dataset identifier PXD044220 (https://www.ebi.ac.uk/pride/ (accessed on 14 October 2023).

### 2.15. Statistical Analysis

Graphs were plotted using GraphPad Prism Software 6 (GraphPad Software, La Jolla, CA, USA). All values are presented as mean ± SEM from at least three independent experiments with suitable number of technical replicates (at least three). A two-tailed Student’s *t*-test was used to estimate the statistical significance between two groups. Significance was set up at *p* ≤ 0.05 (unless stated otherwise).

## 3. Results

### 3.1. CYLD DUB Deficiency Affects Reprogramming Efficiency

**Figure 1:**
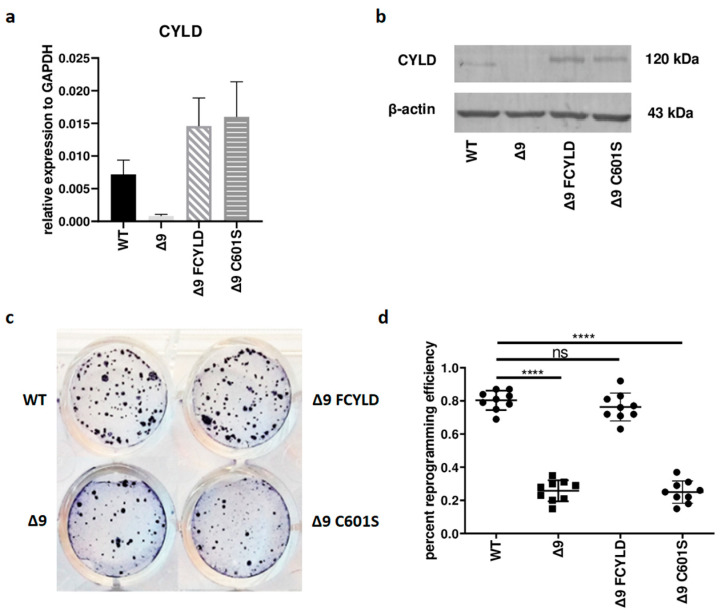
CYLD DUB deficiency reduces reprogramming efficiency. (**a**,**b**) CYLD expression levels in the indicated MEFs (WT, Δ9, Δ9 FCYLD, Δ9 C601S) determined by qPCR (**a**) and immunoblotting (**b**). (**c**) Representative example of ALP-positive staining. CYLD catalytic inactivity leads to the formation of a smaller number of iPSC colonies. (**d**) Cumulative results of ALP staining in WT and Δ9 reprogrammed MEFs of the three established cell lines (b2_1, b2_2 and b3). CYLD DUB deficiency leads to a mean 75% reduction in reprogramming efficiency compared to MEFs expressing the WT *CYLD* (raw ALP staining results of the three MEF cell lines are presented in Figure S4). Data shown are the mean ± SEM from *n* = 3 independent experiments, two-tailed Student’s *t*-test (**** *p* ≤ 0.0001, Abbreviation: ns, not significant). The uncropped blot is shown in Supplementary Figure S3.

Initially, it was tested whether *CYLD* plays any regulatory role in somatic cell reprogramming. For this purpose, WT, Δ9, Δ9FCYLD and Δ9C601S MEFs were reprogrammed into pluripotent stem cells. The expression of CYLD in all cell types used was measured with both qPCR and Western blot (Figure 1a,b) (the uncropped blot is presented in Figure S3). At the end of the reprogramming period (day 18), cells were stained for Alkaline Phosphatase (ALP), which is highly expressed in undifferentiated cells, thus it is used as a surface pluripotency marker [1]. A representative example of ALP staining is presented in Figure 1c, while the staining results of each sample can be found in Figure S4. Overall, CYLD DUB deficiency caused a ~75% reduction in reprogramming efficiency compared to WT MEFs (mean efficiency = 0.8% for MEFs carrying the WT *CYLD* and 0.21% for cells carrying a catalytically inactive *CYLD*). Importantly, the introduction of WT *CYLD* in Δ9 MEFs was able to rescue the reprogramming efficiency, which became comparable to that of WT MEFs. On the other hand, the C601S variant had no effect (Figure 1c,d). This indicates that the observed phenotype is caused solely by CYLD catalytic inactivation.

### 3.2. Δ9 CYLD Does Not Affect iPSC Pluripotency

**Figure 2:**
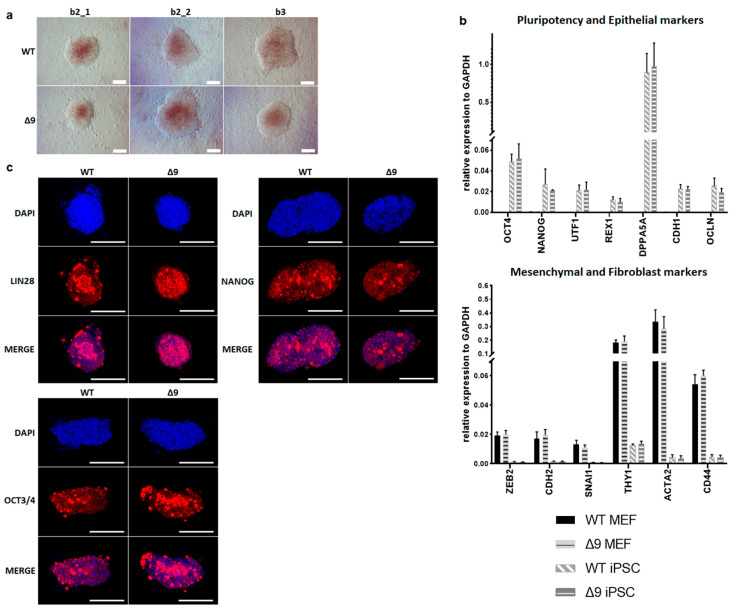
CYLD is dispensable for pluripotency maintenance. (**a**) Bright-field microscopy images of representative iPSC monoclonal colonies after 10 consecutive passages (scale bar: 100 μm). Three WT and Δ9 monoclonal cell lines are presented, derived from the three WT and Δ9 MEF established cell lines (b2_1, b2_2 and b3). (**b**) Relative quantitation by qPCR of pluripotency (*OCT3/4*, *NANOG*, *UTF1*, *REX1*, *DPPA5A*), epithelial (*CDH1*, *OCLN*), mesenchymal (*ZEB2*, *CDH2*, *SNAI1*) and fibroblast (*THY1*, *ACTA2*, *CD44*) markers in the three WT and Δ9 monoclonal iPSC cell lines described in (**a**). (**c**) Representative immunofluorescence staining of the pluripotency markers *OCT3/4*, *NANOG* and *LIN28* in WT and Δ9 iPSCs (scale bar: 100 μm). Data shown are the mean ± SEM from *n* = 3 independent experiments.

Despite the reduction observed in cell reprogramming efficiency, Δ9 MEFs formed colonies of apparent reprogrammed cells. Consequently, it was tested whether Δ9 iPSCs are fully pluripotent. For this purpose, WT and iPSC colonies were harvested on day 18 and established stable, monoclonal iPSC cell lines from all WT and Δ9 MEF cell lines reprogrammed. iPSC cultures were maintained in DOX-free conditions. After 10 consecutive passages, Δ9 iPSC exhibited an indistinguishable morphology to control iPSCs (Figure 2a) and expressed comparable, high levels of core (*OCT3/4*, *NANOG*) and late (*DPPA5A*, *REX1*, *UTF1*) [17,116,117] pluripotency markers, as well as epithelial markers (*CDH1*, *OCLN*) [118,119] (Figure 2b). Additionally, in both iPSC types, mesenchymal (*CDH2*, *SNAI1*, *ZEB2*) [118,119] and fibroblast markers (*ACTA2*, *CD44*, *THY1*) [120,121] were expressed in comparable levels and presented a dramatic decrease compared to MEFs (Figure 2b). Furthermore, both iPSC types expressed comparable, low levels of *T* and *EOMES*, which are markers of primed pluripotency and incomplete reprogramming [122] (Figure S5a). The protein expression of pluripotency markers was further investigated with immunofluorescence. It was observed that both the expression and localization of pluripotency markers OCT3/4, NANOG and LIN28 were comparable in WT and Δ9 iPSC colonies. In conclusion, CYLD inactivation does not affect the pluripotency maintenance of iPSCs.

### 3.3. CYLD Is Dispensable for In Vitro Spontaneous Differentiation

A hallmark of pluripotency is the ability of stem cells to spontaneously differentiate into cells from the three embryonic germ layers. Therefore, it was tested whether CYLD DUB deficiency could potentially affect this process. For this purpose, WT and Δ9 iPSCs derived from the three independent MEF cell lines were cultured on suspension, under LIF-free conditions. After 3 days, both cell types formed Embryoid Bodies (EBs) of similar morphology (Figure S5b). Since the differentiation process starts with the formation of EBs, it was tested whether the downregulation of pluripotency genes followed a similar pattern in WT and Δ9 cells. It was observed that both cell types presented comparable downregulation rates (Figure S5c). On day 3, EBs were plated on gelatin-coated plates and cultured on FBS-containing medium. On the 14th day of culture, both cell types expressed comparable levels of endodermic (*AFP*, *GATA4*), mesodermic (*ACTA2*, *BRACHYURY*) and ectodermic (*NESTIN*, *PAX6*) markers [123,124] (Figure S5d). It was then investigated whether CYLD DUB deficiency could affect the kinetics of differentiation. To this end, cells were allowed to differentiate spontaneously and sampled from days 0-4, 7, 10 and 14 to assess the downregulation rate of pluripotency genes (*OCT3/4*, *NANOG*) and the upregulation of developmental genes (Endoderm: *AFP*, *GATA4*; Mesoderm: *ACTA2*, *BRACHYURY*; Ectoderm: *NESTIN*, *PAX6*; Trophoectoderm: *EOMES*). In both cases, the tested cell types exhibited comparable behavior. In conclusion, CYLD DUB deficiency does not affect the spontaneous differentiation potential of iPSCs (Figure S6).

### 3.4. CYLD Regulates Early Reprogramming

**Figure 3:**
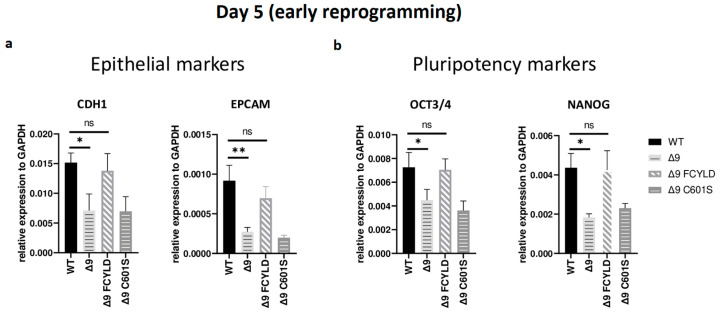
*CYLD* regulates early reprogramming. qPCR-based relative quantitation of epithelial (*CDH1*, *EPCAM)* (**a**) and pluripotency (*OCT3/4*, *NANOG*) (**b**) genes on day 5, which corresponds to the early (initiation) phase of reprogramming. Tested mesenchymal markers for this day are presented in Figure S7b. Harvested day 5 early iPSC colonies were derived from reprogrammed WT and Δ9 MEFs of the three established MEF cell lines (b2_1, b2_2 and b3) and MEFs from the three independent Δ9 FCYLD and Δ9 C601S derivative cell lines. Data shown are the mean ± SEM from *n* = 3 independent experiments for each cell line, two-tailed Student’s *t*-test (* *p* ≤ 0.05, ** *p* ≤ 0.01. Abbreviation: ns, not significant **).

It has been firmly established that somatic cell reprogramming can be separated into three distinct phases: initiation (or early reprogramming), maturation (intermediate) and stabilization (late) [17,63]. Aiming to identify which phase (or phases) is mostly affected by CYLD DUB deficiency, early iPSC colonies derived from the three independent MEF cell lines were harvested from specific days, which are representative of each of the three phases—5 for early, d10 for intermediate and d16 for late reprogramming. Colonies were manually harvested and chosen based on their morphological characteristics (Figure S7a). More specifically, clusters of cells with cuboidal morphology (early iPSCs) [125] and compact colonies with distinct borders and well-defined edges (late iPSCs) [126] were chosen. The expression of markers specific to each phase was measured with qPCR. Both cell types exhibited similar expression patterns on days 10 and 16 (Figure S7b,c). Of particular importance for these phases are *SOX2* and *ESRRB* since they are activated during the maturation phase [63,127]. In a similar manner, the expression of late pluripotency markers *GBX2* [128], *DPPA5A* [117], *UTF1* [17] and *REX1* [116] follows the same trend in all cases. However, CYLD DUB-deficient cells presented lower expression levels of epithelial genes, such as *CDH1* and *EPCAM* (Figure 3a)*,* which are considered early reprogramming markers [17,63,118]. The downregulation of mesenchymal and somatic (fibroblast) genes is another key event of early reprogramming [17,63,118]; however, at day 5, no statistically significant difference in the expression of said markers (fibroblast: *TGFB3*, *THY1* [120,127]; mesenchymal: *SNAI1*, *ZEB2* [118,119]) between CYLD DUB-deficient and WT cells was detected (Figure S7b). On the other hand, CYLD DUB-deficient early iPSCs expressed lower levels of the core pluripotency genes *OCT3/4* (endogenous) and *NANOG* compared to their WT counterparts (Figure 3b). This indicates that CYLD affects somatic cell reprogramming by regulating the initiation phase. 

### 3.5. CYLD DUB Deficiency Delays Early Reprogramming Progression

Early reprogramming is a complex and multifaceted process in which many mechanisms are involved. Among the better-studied steps are the mesenchymal-to-epithelial transition (MET), the glycolytic shift, the loss of somatic cell identity and the increase in proliferation rates [20,63]. Interestingly, there are two distinct events observed during early reprogramming. The first days of reprogramming are characterized by elevated levels of mesenchymal genes, such as *CDH2*, *SNAI1* and *ZEB2,* followed by the activation of epithelial and epidermal genes, like *CDH1*-*OCLN* and *KRT14*-*KRT17*, respectively, and the concomitant downregulation of mesenchymal and somatic genes [120,129,130]. This set of changes is considered a hallmark of MET [118,131]. Based on this, it has been suggested that the initiation phase can be split into two stages, a mesenchymal one, which takes place during the first days after the expression of the reprogramming cassette, followed by an epidermal stage [19]. Based on previous studies with the same reprogramming protocol [110], MET starts at day 3, when the first early iPSC colonies become observable, whereas the initiation phase lasts until day 6, when cells are plated onto feeders.

In order to better understand the way in which CYLD DUB deficiency affects these processes, early iPSC colonies from the three independent MEF cell lines were harvested during the initiation phase, and the relative expression of markers characteristic to each event was measured. High-quality and without fibroblast contamination, early iPSC colonies from all days considered to be part of the second stage of early reprogramming (D3–D6) were harvested (phenotypic characteristics of these colonies are described in Figure S8a). CYLD DUB-deficient cells express lower levels of core pluripotency and epithelial genes from the start and throughout the initiation phase. However, on day 6, the expression levels were on par with WT and Δ9 FCYLD early iPSCs. On the other hand, the mesenchymal and somatic genes tested exhibited similar expression patterns and were adequately downregulated by day 3 (Figure S8c).

**Figure 4:**
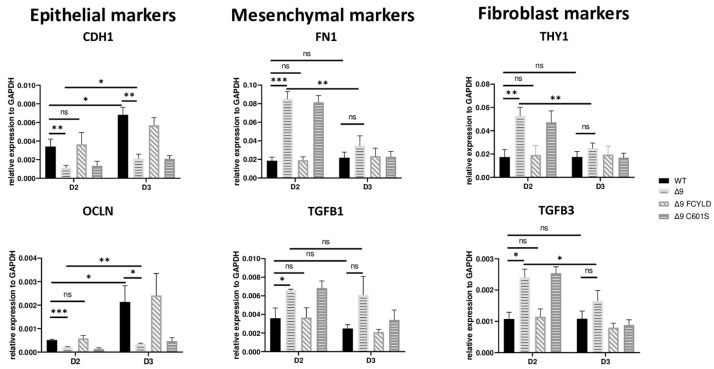
CYLD is required for MET activation. qPCR-based relative quantitation of the indicated epithelial, mesenchymal and fibroblast markers in the indicated cell types on day 2 of the reprogramming process. Harvested D2 and D3 early iPSCs were derived from reprogrammed WT and Δ9 MEFs of the three established cell lines (b2_1, b2_2 and b3) and MEFs from the three independent Δ9 FCYLD and Δ9 C601S derivative cell lines. Data shown are the mean ± SEM from *n* = 3 independent experiments for each cell line, two-tailed Student’s *t*-test (* *p* ≤ 0.05, ** *p* ≤ 0.01, *** *p* ≤ 0.001, Abbreviation: ns, not significant).

These results indicate that CYLD DUB deficiency impedes early reprogramming progression. To test this hypothesis, we harvested samples from day 2, a transitional state before the initiation of MET, where cells alter their shape and are clustered together but have not yet formed pre-iPSC colonies (Figure S8a). Indeed, cells lacking catalytically active CYLD express higher levels of both mesenchymal (*FN1*, *TGFB1*) [119,132] and somatic markers (*TGFB3*, *THY1*) [120,127]. On par with that, the expression levels of epithelial genes (*CDH1*, *OCLN*) [119] are lower compared to WT and Δ9 FCYLD cells (Figure 4). Interestingly, even though CYLD DUB-deficient cells managed to successfully downregulate the mesenchymal and somatic markers tested by day 3, this was not the case with the epidermal and epithelial markers, which remained at notably lower levels compared to control cells. Taken together, CYLD DUB deficiency affects early reprogramming by hindering the progression of MET. All studied genes can be found in Figure S8d.

### 3.6. CYLD Regulates MET and ECM Organization

**Figure 5:**
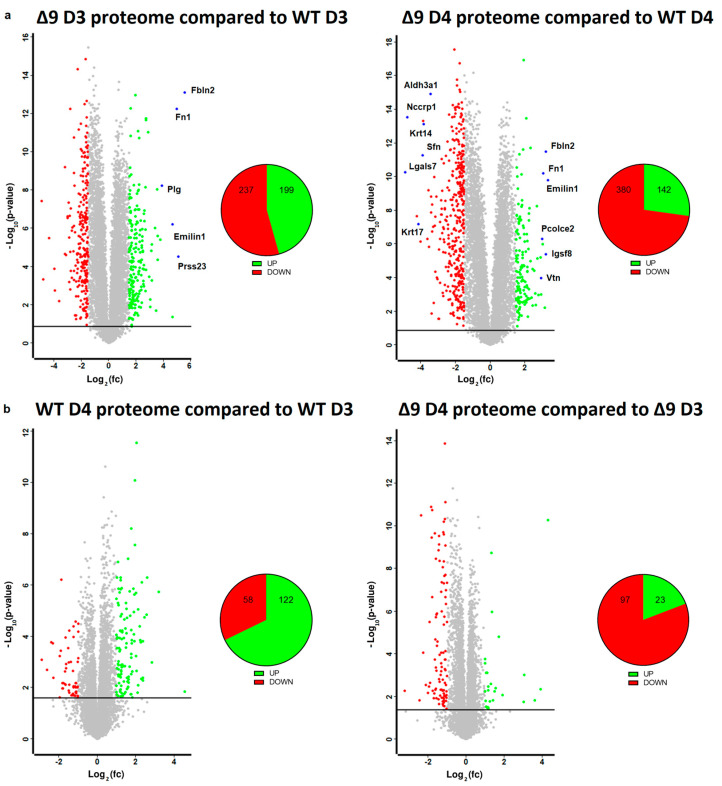
Whole proteome profiling of D3 and D4 early iPSCs. (**a**) Volcano plots (FDR = 0.05, S0 = 0) comparing the Δ9 early iPSCs proteome to that of WT early iPSCs (D3: left plot, D4: right plot). A threshold of |log_2_fc ≥ 1.5| and log_2_ (*p*-value) ≥ 1.5 is applied to determine the statistically significant Differentially Expressed Proteins (DEPs). Proteins downregulated in Δ9 samples compared to WT samples are marked as red, whereas upregulated proteins are marked as green. Proteins of interest are highlighted in blue. CYLD DUB deficiency led to the upregulation of 199 and downregulation of 237 proteins compared to WT day 3 early iPSCs. Similarly, 142 proteins were upregulated and 380 downregulated compared with WT samples on day 4. At both time points, some of the most overexpressed proteins in Δ9 early iPSCs are associated with the extracellular matrix, whereas epithelial, epidermal and early reprogramming markers are underrepresented. (**b**) Volcano plots (FDR = 0.05, S0 = 0) comparing the proteome of D4 early iPSCs proteome to that of D3 early iPSCs (WT: left plot, Δ9: right plot). A threshold of |log_2_fc ≥ 1| and log_2_ (*p*-value) ≥ 1.5 is applied to determine the statistically significant DEPs. D3 and D4 harvested early iPSCs are derived from WT and Δ9 MEFs of the three established cell lines (b2_1, b2_2 and b3). Each sample was analyzed in at least two technical replicates.

Based on the results mentioned above, it was clear that CYLD DUB deficiency compromises the delicate balance that leads to MET activation. In order to gain insight into the way CYLD DUB deficiency disrupts the early reprogramming network, WΤ/Δ9 D0 MEFs of the three independent cell lines and the D3–D4 early iPSC colonies (of said cell lines) were harvested and subjected to whole proteome analysis through LC/MS. Day 3 proteome corresponds to the transcriptome of day 2 samples and day 4 proteome to that of day 3 samples. A total of 7163 proteins were identified (the z-score of identified proteins is given in Table S2). On D4, there were 5368 statistically significant Differentially Expressed Proteins (DEPs) in Δ9 early iPSCs compared to WT (2647 upregulated and 2721 downregulated). Similarly, on D3, there were 4910 DEPs in Δ9 early iPSCs (2347 upregulated and 2562 downregulated). After applying a threshold of |log_2_fc ≥ 1.5| and log_2_ (*p*-Value) ≥ 1.5, the number of DEPs dropped to 436 for D3 (199 upregulated and 237 downregulated) and 522 for D4 (142 upregulated and 380 downregulated) (Figure 5a). It was observed that the most overexpressed proteins in Δ9 early iPSCs are associated with the extracellular matrix, as identified by the use of the MatrisomeDB database and the literature research (Emilin1, Fbln1, Fbln2, Fn1, Igsf8, Pcolce2, Plg, Prss23, Vtn) [133,134,135]. On the other hand, markers of early reprogramming as well as epithelial and epidermal proteins associated with MET (Aldh3a1, Lgals7, Nccrp1, Sfn, Krt14, Krt17) [120,130,136,137,138] were downregulated (blue dots).

Due to the low number of DEPs between the D3–D4 transition, a less strict threshold of |log_2_fc ≥ 1| was applied in this analysis. It was revealed that the majority of WT D4 DEPs are upregulated in comparison to D3 iPSCs (122 DEPs upregulated and 58 downregulated), whereas the opposite trend is observed in Δ9 early iPSCs (23 DEPs upregulated and 97 downregulated) (Figure 5b). Both WT and Δ9 MEFs followed a similar pattern during the transition from D0 to D3, with most DEPs being upregulated. However, this effect was more pronounced in Δ9 cells (Figure S9a). Finally, our analysis revealed that CYLD DUB deficiency also affected the proteome of MEFs. More specifically, 311 DEPs were downregulated and 82 upregulated in Δ9 MEFs compared to WT MEFs at day 0 (Figure S9b).

**Figure 6:**
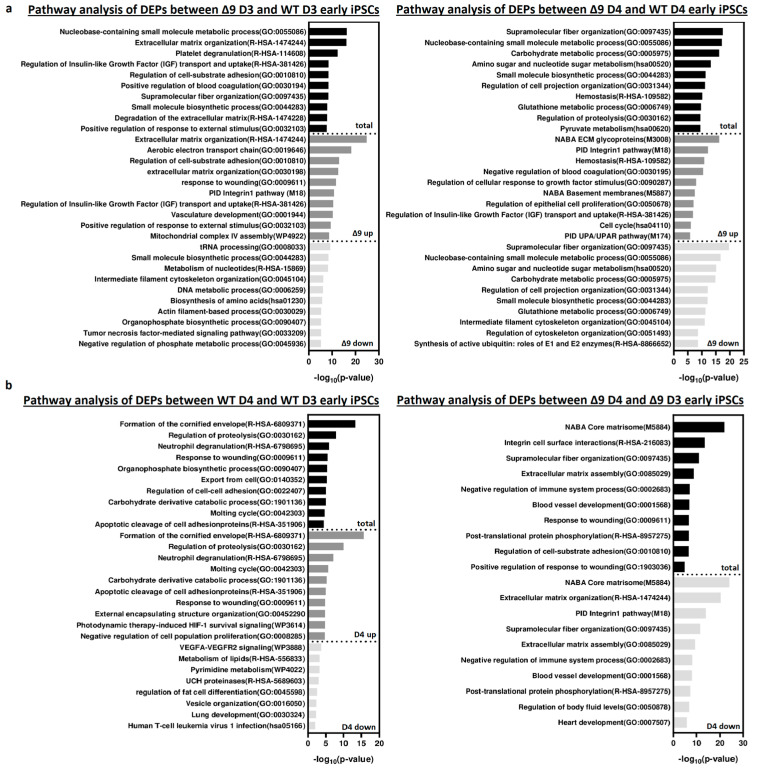
Pathway enrichment analysis of DEPs from D3 and D4 early iPSCs. (**a**) Analysis of DEPs between Δ9 D3 (left) and D4 (right) compared to their WT counterparts. Upregulated DEPs significantly correlate with extracellular matrix organization, cell adhesion and several developmental processes. On the other hand, downregulated DEPs participate in several metabolic processes. (**b**) Analysis of D3–D4 transition DEPs in WΤ (left) and Δ9 (right) early iPSCs. Upregulated DEPs in WT cells are mostly associated with the formation of the cornified envelope and the regulation of proteolysis. Downregulated DEPs are fewer, and the enrichment analysis presented a weak association with some metabolic and developmental processes, the most notable of them being the VEGF signaling pathway. In the case of Δ9 early iPSCs, downregulated DEPs have a strong link with extracellular matrix organization and the integrin 1 pathway. The very small number (23) of upregulated proteins did not present a significant association with any process. Pathway analysis was conducted with Metascape [139].

Pathway enrichment analysis with Metascape [139] revealed that DEPs upregulated in Δ9 cells are strongly correlated with extracellular matrix organization, cell–substrate adhesion and several developmental processes (for example, hemostasis, response to wounding and regulation of blood coagulation) (Figure 6a). Most of the processes overrepresented in Δ9 cells, notably the extracellular matrix organization, cell adhesion and the integrin pathway, are known to be downregulated during the MET, where structural reorganization occurs [130,140]. Indeed, iPSCs express a different set of ECM proteins compared to MEFs [48]. The remodeling of ECM (destruction of MEF-associated ECM and development of iPSC-associated ECM) during reprogramming is paramount for the establishment of the unique iPSC morphology and microenvironment [48,141]. Intriguingly, one of the enriched pathways associated with Δ9 D4 upregulated DEPs is the “Regulation of Epithelial Cell Proliferation Pathway”. However, most of the associated DEPs are associated with EMT progression, so they are expected to play a negative role in epithelial cell identity (Table S3). Conversely, downregulated DEPs are mostly associated with several metabolic processes and cytoskeletal organization. Interestingly, pathway analysis of the D3–D4 transition provided markedly different results for WT and Δ9 cells (Figure 6b). More specifically, upregulated DEPs in WT D4 early iPSCs are primarily associated with the formation of the cornified envelope and regulation of proteolysis. The number of downregulated DEPs was relatively small (58), and as a result, their association with the presented processes is weak (low −log_10_ (*p*-Value)). On the contrary, downregulated DEPs of Δ9 cells presented a strong correlation with extracellular matrix organization and the integrin pathway. The number of upregulated DEPs was very small, so the pathway analysis failed to construct any correlation. Regarding the D0-D3 transition, upregulated DEPs presented many similarities between WT and Δ9 MEFs, with most proteins being associated with the same biosynthetic and metabolic processes (for example, the biosynthesis of cofactors and tRNA, nucleoside monophosphate and amino acid metabolic process). However, the pattern was not the same with the downregulated proteins. In WT cells, downregulated DEPs presented a strong association with the extracellular matrix organization, a process that is completely absent from Δ9 cells. Similarly, processes highly enriched in Δ9 cells, such as vasculature development and the integrin 1 pathway, are not observed in WT cells (Figure S10a). Finally, comparison of D0 MEFs revealed that most downregulated DEPs in Δ9 cells are associated with several metabolic processes, whereas upregulated DEPs presented a weak association. Interestingly, the combined analysis was enriched in several processes associated with the immune response, such as the NF-κB pathway, response to viral infection and macroautophagy (Figure S10b).

**Figure 7:**
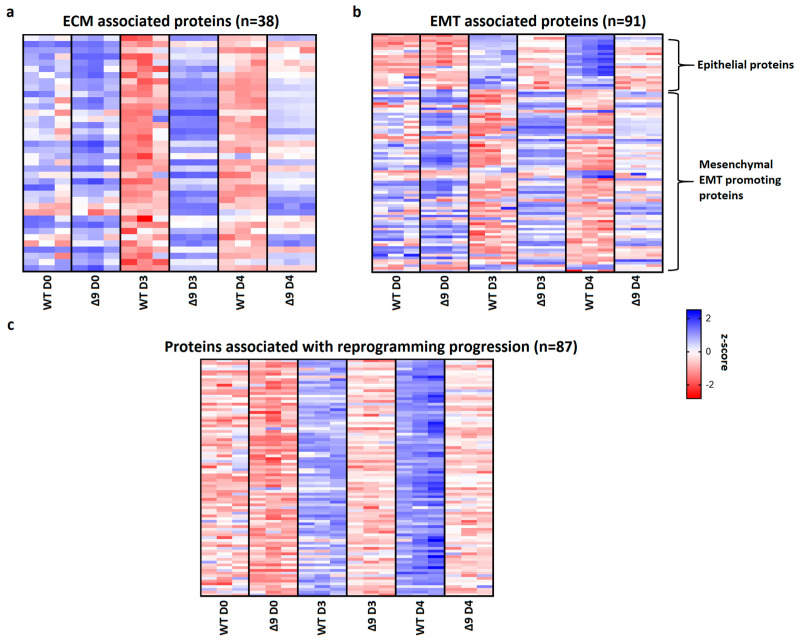
CYLD regulates MET. (**a**) Expression pattern of 38 extracellular matrix-related proteins that were identified from pathway analysis. On both D3 and D4, Δ9 early iPSCs express higher levels of these proteins compared to WT cells, although this is more prominent on D3. (**b**) Expression pattern of 91 identified EMT−related proteins. Epithelial−related proteins are presented on top. CYLD DUB−deficient cells express lower levels of epithelial proteins and higher levels of mesenchymal proteins compared to WT cells. While mesenchymal proteins are slightly downregulated on D4 compared to D3, Δ9 cells were not able to increase the expression of epithelial proteins on D4. (**c**) Expression pattern of 87 identified reprogramming-associated proteins. Activation of these proteins is concomitant to MET initiation. CYLD DUB-deficient cells present vastly lower expression levels of all these proteins compared to WT cells. On the other hand, WT early iPSCs steadily increase most of them on D4 compared to D3. Three independent WT and Δ9 MEF cell lines were used (b2_1, b2_2 and b3, respectively). Harvested D3 and D4 early iPSC colonies are derived from these reprogrammed WT and Δ9 MEF cell lines. Each sample was analyzed in at least two technical replicates (z−scores of DEPs from every sample are presented in Table S2).

Pathway analysis of D3 and D4 samples indicated that a set of 38 extracellular matrix-associated proteins are differentially expressed (Table 1). Notable members of this group are Fn1, Emilin1 and Fbln2, which were shown above to be the most overexpressed proteins in Δ9 cells. A heatmap presenting the expression patterns of the extracellular matrix proteins across the tested samples is available in Figure 7a. As it can be clearly observed, most of these proteins are significantly overexpressed in Δ9 cells on day 3 of reprogramming. Moreover, this trend is also present, although to a lesser extent, on day 4.

Since many of these proteins are also involved in the EMT, and taking under consideration that hallmark epithelial genes such as *CDH1* and *EPCAM* are downregulated in Δ9 cells, the number of D3 and D4 DEPs associated with the EMT was investigated. Through the literature search, the use of the EMTome portal [142] and the Hallmark Epithelial Mesenchymal Transition Gene Set (Molecular Signature Database, MSigDB) [143], 91 EMT-associated DEPs were identified; 21 epithelial and 70 mesenchymal or EMT-promoting DEPs. A heatmap presenting the expression pattern of these proteins on days 0, 3 and 4 is available in Figure 7b.

The expression levels of epithelial proteins are substantially lower in both D3 and D4 compared to WT early iPSCs. At the same time, most mesenchymal and EMT-promoting proteins are upregulated in Δ9 early iPSCs, although it is worth noting that on D4 their expression has decreased. D0 MEFs show comparable expression levels of most proteins. Finally, we identified 87 proteins whose expression is associated with successful reprogramming (Figure 7c), based on previous research [17,19,120,129,130,137,138,140]. Regardless of their different expression patterns throughout reprogramming, these proteins are activated during MET. While all these proteins have been successfully upregulated in Δ9 early iPSCs compared to MEFs, their expression is considerably lower compared to WT early iPSCs, suggesting a lower rate of reprogramming kinetics. To summarize, our proteomic results indicate that Δ9 early iPSCs express higher levels of EMT-associated markers and extracellular matrix proteins, while failing to adequately activate epithelial and MET-associated proteins (the complete list of genes included in Figure 7 heatmaps is available in Table S2).

## 4. Discussion

**Figure 8:**
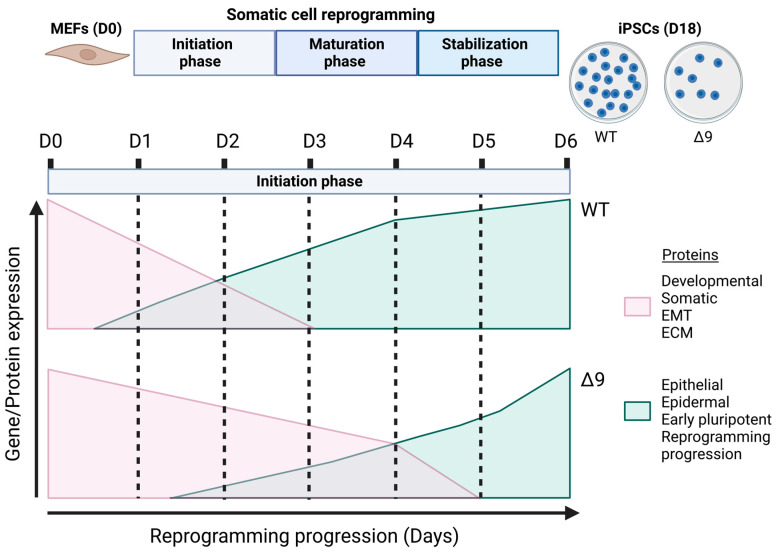
Proposed role of CYLD in somatic cell reprogramming. CYLD DUB-deficient MEFs (*CYLD^Δ9/Δ9^*) present a 75% reduction in reprogramming efficiency compared to WT MEFs (*CYLD^WT/WT^*), as determined through ALP staining. Gene profiling and whole proteome analysis identified the initiation phase of reprogramming as mostly affected by CYLD DUB deficiency, hindering early reprogramming progression. Δ9 early iPSCs are characterized by higher expression levels of developmental, somatic (fibroblast), EMT- and ECM-associated proteins (which act as reprogramming barriers) and lower expression levels of epithelial, early pluripotency and reprogramming-progression-associated proteins. As a result, the process of MET is severely affected (this figure was generated with BioRender, https://www.biorender.com/). Abbreviations: D= day of reprogramming.

The current study revealed for the first time the regulatory role of CYLD in somatic cell reprogramming. MEFs lacking a catalytically active protein presented lower reprogramming efficiency and slower early reprogramming kinetics compared to WT MEFs, with the transition from the first to the second stage of the initiation phase being most affected (Figure 8). However, it is worth noting that CYLD DUB-deficient cells were able to be fully reprogrammed. Δ9 iPSCs were stable after 10 consecutive passages and presented similar phenotypic and molecular characteristics to WT iPSCs. Moreover, they could spontaneously differentiate into cells of the three germ layers and followed the same differentiation kinetics pattern as WT cells. It would be very interesting to investigate whether this is the case with directed differentiation also, especially towards cell types that are known to be negatively affected by CYLD inactivation, such as hepatocytes [91,144,145,146], cardiomyocytes [147,148,149,150] and neurons [151,152,153,154].

The mechanism by which CYLD DUB deficiency slows down the transition to the second stage of initiation phase is likely the obstruction of MET through the promotion of the opposite process, EMT. As our proteomics results demonstrated, CYLD DUB-deficient cells are characterized by the aberrant upregulation of mesenchymal and EMT-promoting proteins and low expression of epithelial proteins, in contrast to WT early iPSCs. This correlates with the inadequate activation of proteins associated with successful reprogramming. As previously stated, these proteins are also markers of the MET since they are activated during that process [120,130,140]. These results strengthen our early reprogramming kinetic experiments, where we demonstrated that CYLD DUB-deficient cells activate epithelial and pluripotency genes at a lower rate. Indeed, analysis of the D3-D4 transition supports this observation. In more detail, the main difference between WT D3 and D4 early iPSCs is the upregulation of MET- and reprogramming-associated proteins. On the other hand, Δ9 D4 early iPSCs are still in the process of downregulating mesenchymal and ECM-associated genes, as the pathway analysis of D3–D4 transition revealed.

The reason behind the EMT-promoting phenotype of Δ9 cells is not clear yet. As previously mentioned, the first transcriptional wave observed during somatic cell reprogramming is characterized by the downregulation of somatic and mesenchymal genes, along with genes associated with several developmental processes, such as vasculature, bone and heart development [120,129,130,140]. The major event of this stage (which is also called the mesenchymal stage [19]) is the downregulation of Thy1 [120,129] and roughly corresponds to the first day of our reprogramming model. The absence of Thy1 from the cell surface signifies the end of this stage [120,129]. Whether EMT- and ECM-associated genes are hyperactivated in Δ9 cells at this stage or their downregulation follows a slower rate is not known. Since cells at D1 cannot be distinguished from their surroundings nor can they be individually harvested with our protocol, we were unable to isolate pure samples from this stage. However, it is worth mentioning that pathway analysis of the Δ9 D0-D3 transition did not include the process of extracellular matrix organization, which was the primary downregulated event of the WT D0-D3 transition. Furthermore, the proteomic results suggest that a number of key mesenchymal stage players, such as Tgf-β1 and Fn1, presented a small but statistically significant upregulation in Δ9 D3 early iPSCs compared to MEFs. While these observations likely point out to an aberrant activation of EMT during D1, other proteins, including Thy1 and Thbs1, are significantly downregulated in Δ9 D3 iPSCs compared to Δ9 MEFs. As a result, no safe conclusion can be made based on the current results, and further research on D1 cells is required. Regardless, the overexpression of EMT genes/proteins and several components of the TGF-beta pathway (such as *TGFΒ1*, *TGFBi*, *LTBP1* and *THBS1*) is consistent with previous observations that associate CYLD DUB deficiency with TGF-beta activation and EMT promotion [85,86,87,108,155], although it is not yet known whether the underlying mechanism is the same in the case of somatic cell reprogramming.

Importantly, the negative effect of CYLD DUB deficiency on epithelial and epidermal gene activation is further supported by the transcriptomic analysis of WT and Δ9 D3 early iPSCs (data not shown). Importantly, 17 out of the 21 identified epithelial DEPs and several proteins associated with successful reprogramming progression showcased high correlation with the transcriptomic results, further supporting the findings of the present study.

A finding of significant importance is the ability of CYLD to regulate the processes of extracellular matrix organization, which is proposed for the first time in this study. This can have a considerable implication in cancer research, since this process is known to play a critical role in cancer development, growth and invasion [156,157,158,159]. Regarding its role in somatic cell reprogramming, several studies [48,120,130,140] have demonstrated that the downregulation of ECM genes is a hallmark of the MET and failure to do so is associated with poor reprogramming efficiency. Indeed, the ECM is a driver of the EMT [160], so the hyperactivation of ECM-related proteins in Δ9 cells may contribute independently to MET obstruction. 

Another point worthy of discussion is the potential involvement of Irf6. Our results revealed that CYLD DUB-deficient cells failed to activate Irf6 during the first days of reprogramming (Table S2). It has been recently shown that irf6 is a member of a nine-transcription-factor (TF) complex that plays a key regulatory role in early reprogramming, facilitating the transition to the next phase [110]. More specifically, early iPSC colonies that express all nine transcription factors simultaneously have significantly higher chances of becoming fully reprogrammed. Interestingly, RNAi knockdown of Irf6 reduced reprogramming efficiency by 60%. It was found that the expression of Irf6 (along with Gli2 and Ovol1, two other components of the 9TF assembly) is induced between days 2 and 3 by mostly stochastic events related to the OSKM reprogramming cassette. However, it is highly likely that the processes triggered by CYLD DUB deficiency might interfere with these mechanisms.

Besides EMT and ECM regulation, our pathway analysis revealed that CYLD DUB deficiency negatively affects various metabolic processes (such as glutathione, nucleotide and carbohydrate metabolism) not only during reprogramming but also in MEFs. While it is known that metabolic changes play a crucial part in reprogramming, especially the metabolic shift from oxidative phosphorylation to glycolysis [161,162], this process was not observed in our pathway analysis. While further research is needed to identify the effect of CYLD DUB deficiency in these metabolic processes, one valid hypothesis is that these changes correlate with the promotion of EMT, as it has been demonstrated in cancer development [64,163]. One potential way to uncover the role of the CYLD-affected metabolic processes in somatic cell reprogramming is the study of the differential metabolites between WT and Δ9 early iPSCs [164].

## 5. Conclusions

The present study uncovered for the first time the effect of *CYLD* in somatic cell reprogramming. CYLD is part of the mechanism overseeing a successful mesenchymal-to-epithelial transition during early reprogramming and the successful establishment of pluripotency. Indeed, loss of CYLD promotes the EMT and slows down the progression of early reprogramming, leading to efficiency reduction. Nevertheless, CYLD DUB-deficient cells present self-renewal and differentiation abilities comparable to those of control iPSCs.

## Figures and Tables

**Table 1:** Alphabetical list of upregulated extracellular matrix proteins in Δ9 D3 and D4 early iPSCs. Proteins were identified from the pathway enrichment analysis (extracellular matrix organization, R-HSA-1474244) (Figure 6a). The detailed expression patterns are given in Figure 7 and Table S2.

**Upregulated ECM-associated proteins in Δ9 D3 and D4 early iPSCs**				
Bmp1	**Emilin1**	**Igfbp7**	**Nid1**	**Tgfbi**
Ccn2	Emilin2	Loxl2	Npnt	Thbs1
Ccn4	Fbln1	Loxl4	Pcolce2	Tinagl1
Cd44	Fbln2	Ltbp1	Plg	Tll1
Col16a1	Fbn1	Ltbp2	Sdc2	Tnc
Col18a1	Fbn2	Mfap2	Serpine1	
Col8a1	Fn1	Mfge8	Slit3	
Efemp2	Hspg2	Mxra7	Tgfb1	

## Data Availability

The data presented in this study (and Supplementary Materials) and available upon request. Project Name: Inactivation of tumor suppressor CYLD inhibits fibroblast re-programming to pluripotency. Project accession: PXD044220. The raw proteomic data can be found in the proteomics identification database (PRIDE): https://www.ebi.ac.uk/pride/ (accessed on 14 October 2023) [115]. Project Name: Inactivation of tumor suppressor CYLD inhibits fibroblast re-programming to pluripotency. Project accession: PXD044220.

## Supplementary Materials

The following supporting information can be downloaded at https://www.mdpi.com/article/10.3390/cancers15204997/s1, Figure S1: Transduction efficiency test, Figure S2: Colony harvesting example, Figure S3: Uncropped Western blot from Figure 1b, Figure S4: Total ALP staining results, Figure S5: CYLD DUB deficiency does not affect iPSC pluripotency, Figure S6: Kinetics of differentiation, Figure S7: Kinetics of reprogramming, Figure S8: Kinetics of early reprogramming, Figure S9: Whole proteome profiling of D0 MEFs and D0-D3 transition, Figure S10: Pathway analysis of D0 MEFs and D0-D3 transitions, Table S1: Primer sequences used for qPCR, Table S2: Heatmap gene expression analysis, Table S3: Genes involved in pathway analysis described in Figure 6, Table S4: Genes involved in pathway analysis described in Figure S10.
