# Supplementary material for: Inactivation of Tumor Suppressor CYLD Inhibits Fibroblast Reprogramming to Pluripotency
Source: Cancers (Basel). 2023 Oct 15;15(20):4997. doi: 10.3390/cancers15204997 (PMC10605754; doi:10.3390/cancers15204997)
Supplement: Supplementary file 1 [file cancers-15-04997-s001.zip › supplementary figures and Table S1.pdf]

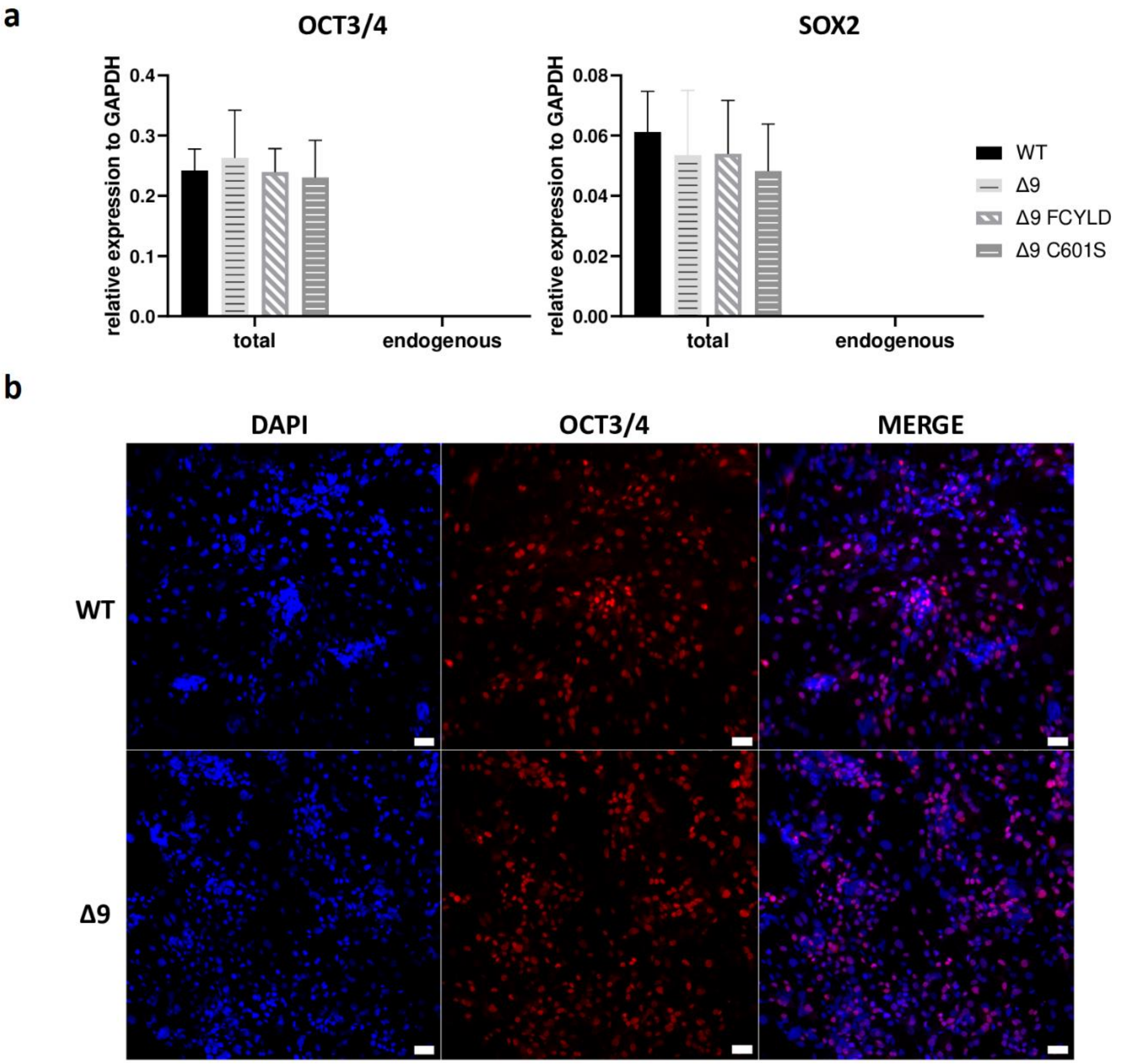

Figure S1. Transduction efficiency test. (a) 24h after the addition of DOX in the medium (day 1 of reprogramming), the expression of total (transgene + endogeous) and endogenous *OCT3/4* and *SOX2* was measured via qPCR. Since the expression of those core pluripotency genes has not been triggered yet at this reprogramming point, total *OCT3/4* and *SOX2* expression measured accounts only to the expression of the reprogramming cassette. To safeguard the reproducibility and validity of our results, we ensured that all reprogramming experiments were carried out with similar transduction efficiency. All experiments were conducted with a transduction efficiency of $0{,}18 \leq 2^{-(\Delta Ct\ OCT3/4)} \leq 0{,}34$ and $0{,}055 \leq 2^{-(\Delta Ct\ SOX2)} \leq 0{,}10$, which roughly corresponds to 1 cycle difference in gene expression. Even though higher transduction efficiency could be achieved, the aforementioned ratio was the one most commonly observed and the one that was eventually chosen as a baseline for our experiments. For the transduction efficiency experiments, WT and Δ9 MEFs of the three established cell lines (b2_1, b2_2 and b3) along with MEFs from the three independent derivative Δ9 FCYLD and Δ9 C601S cell lines were used. Data shown are the mean ± SEM from *n* = 3 independent experiments for each cell line (b) The ratio of infected vs uninfected

MEFs after treatment with the infection cocktail was assessed by immunofluorescence. At day 1 of reprogramming, cells were tested for *OCT3/4* expression. As it can be observed from the representative images, Oct3/4 expression is nearly universal in both cell types, indicating that the infection efficiency was similar in all cases (scale bar: 50μm).

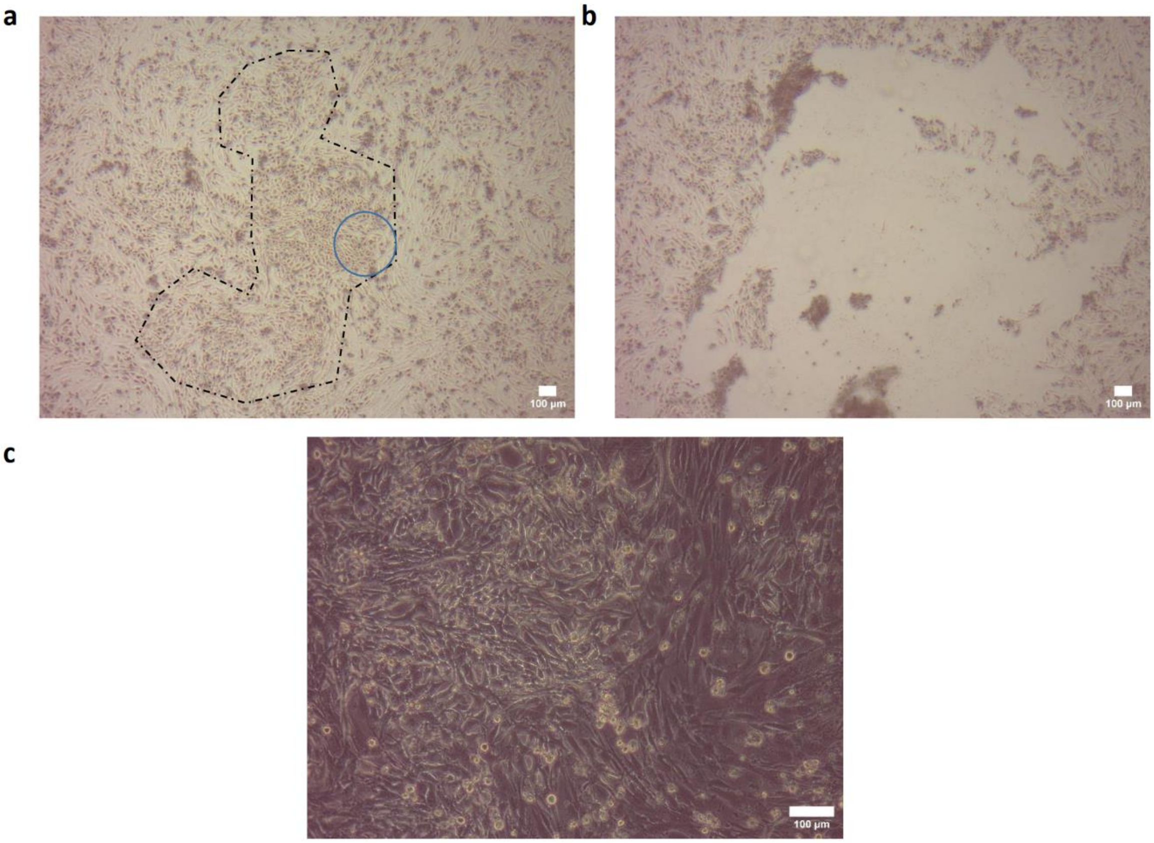

Figure S2. Colony harvesting example. (a) Representative example of a day 3 early iPSC colony. The borders of the colony are marked with a dashed line. (b) after the initial incision across the colony border, the colony is separated from the surrounding area and collected with a pipette. (c) magnification of the area highlighted in the blue circle, showcasing the distinction between the partially reprogrammed cells of the early iPSC colony and surrounding MEFs

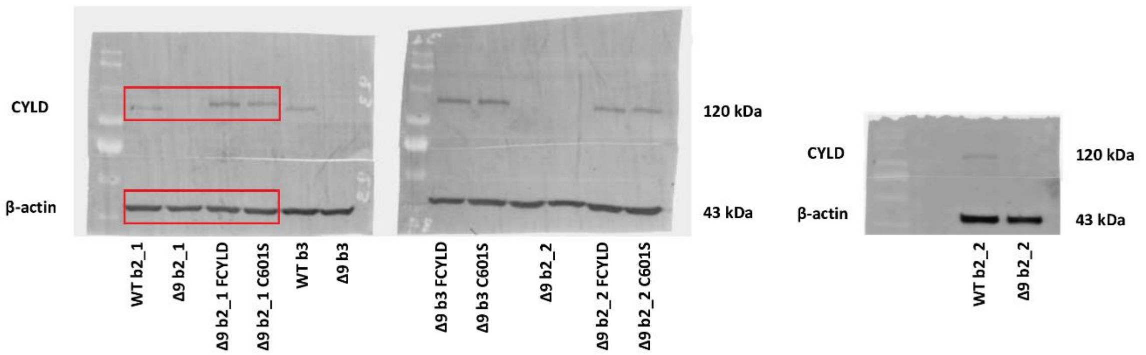

Figure S3. Uncropped western blot from Fig. 1b. The expression of CYLD in the three established WT and Δ9 MEF cell lines (b2_1, b2_2, b3) and the three Δ9 FCYLD and Δ9 C601S derivative cell lines is depicted. The bands presented in the main text are included inside the red box and represent MEF samples from the b2_1 cell line. The second WT sample from the b2_2 cell line was accidentally skipped from the first blot, so a second western blot was conducted which included only the b2_2 WT and Δ9 samples and is presented on the right. It is worth noting that in both Δ9 FCYLD and C601S cell types, the CYLD variant is fused with the FLAG protein, hence the small difference in molecular weight compared to WT CYLD

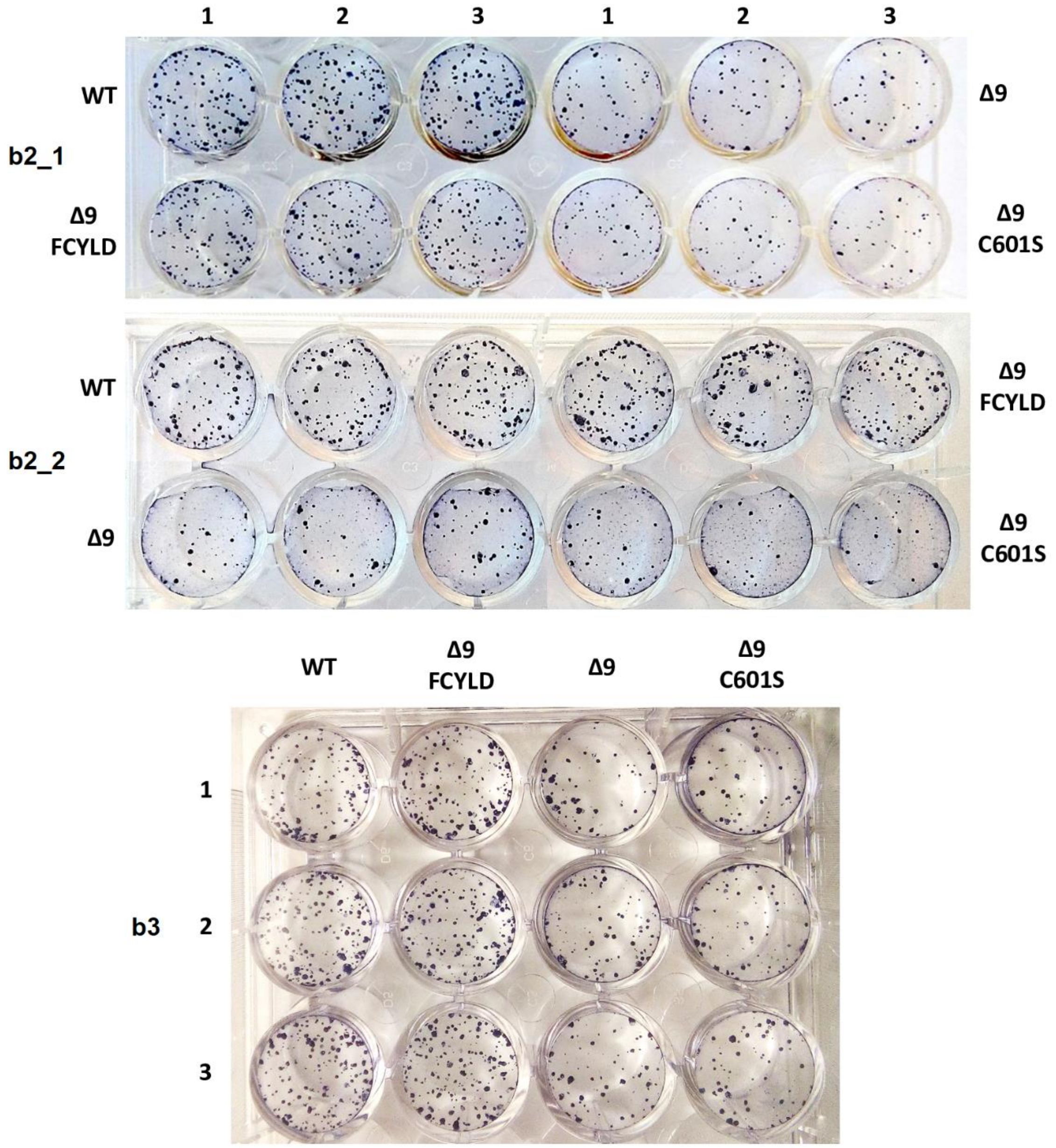

Figure S4. Total ALP staining results. WT and Δ9 MEFs of the three established cell lines (b2_1, b2_2 and b3) along with MEFs from the three independent Δ9 FCYLD and Δ9 C601S derivative cell lines were successfully reprogrammed. For each sample, *n*=3 independent reprogramming experiments were conducted.

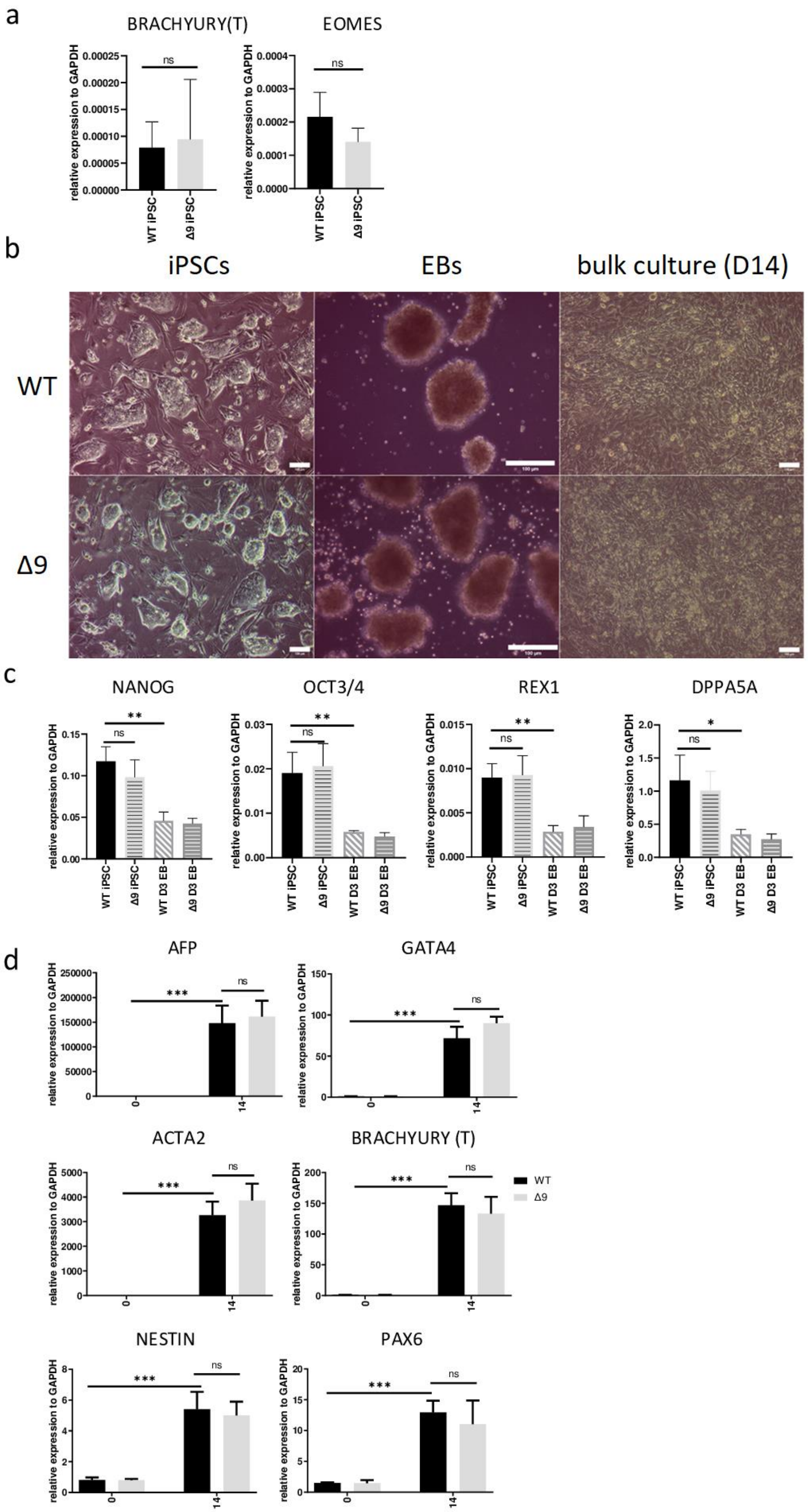
a
BRACHYURY(T)
EOMES
relative expression to GAPDH
ns
WT iPSC
Δ9 iPSC
b
iPSCs
EBs
bulk culture (D14)
WT
Δ9
c
NANOG
OCT3/4
REX1
DPPA5A
relative expression to GAPDH
ns
**
*
WT iPSC
Δ9 iPSC
WT D3 EB
Δ9 D3 EB
d
AFP
GATA4
ACTA2
BRACHYURY (T)
NESTIN
PAX6
relative expression to GAPDH
***
ns
WT
Δ9
0
14

Figure S5. CYLD DUB deficiency does not affect iPSC pluripotency. (a) The expression of developmental, primed pluripotency markers is low and comparable between WT Δ9 iPSCs, indicating naïve pluripotency. (b) Representative images (scale bar: 100μm) of WT and Δ9 cells during key points of spontaneous differentiation (iPSCs-EBs at day 3-14th day of differentiation). (c) The decrease in the expression of core and late pluripotency markers after the formation of EBs is similar between WT and Δ9 cells. (d) After culturing EBs in differentiation medium for 14 days, both cell types managed to successfully differentiate into cells from the 3 germ layers, as indicated by the expression of endodermal (*AFP*, *GATA4*), mesodermal (*ACTA2*, *BRACHYURY*) and ectodermal (*NESTIN*, *PAX6*) markers, as measured via qPCR. No difference in the expression levels of tested markers was observed between WT and Δ9 cells. Every experiment was carried out in iPSCs derived from WT and Δ9 MEFs of the three established cell lines (b2_1, b2_2 and b3). Data shown are the mean ± SEM from n = 3 independent experiments, two-tailed Student's t-test (*$p \leq 0.05$, **$p \leq 0.01$, ***$p \leq 0.001$. Abbreviation: ns, not significant).

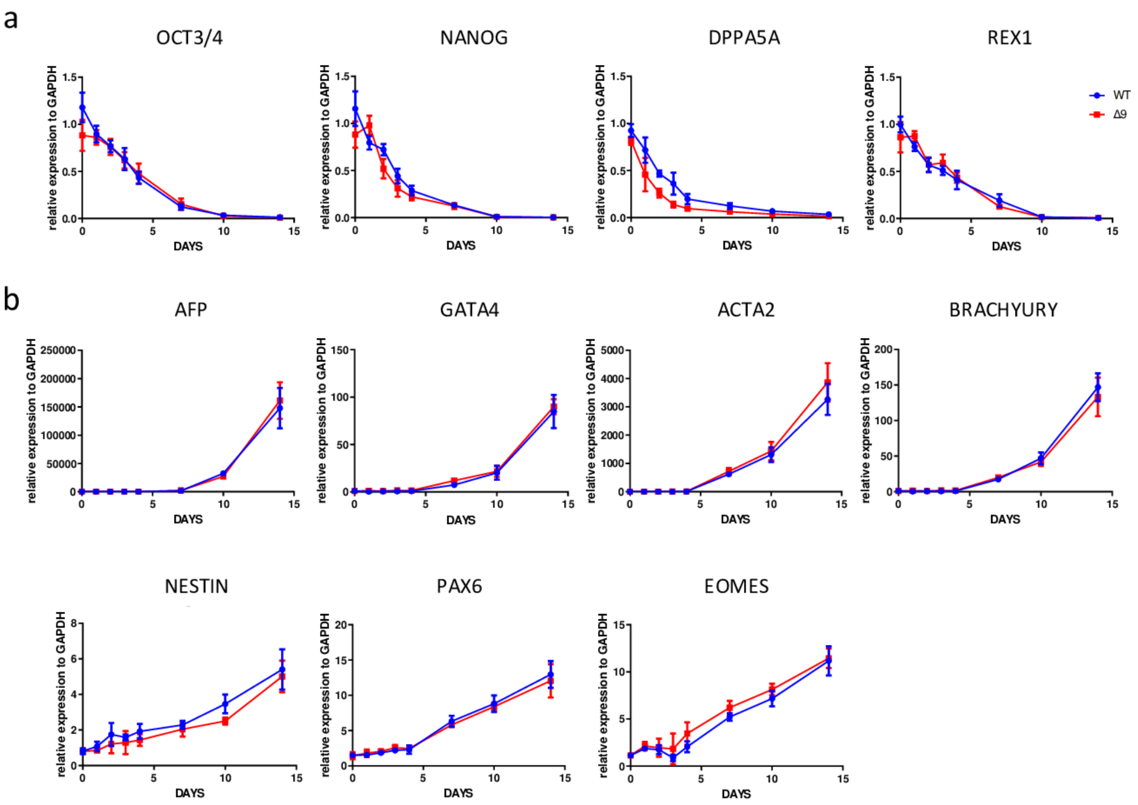

Figure S6. Kinetics of differentiation. (a) The decrease rate of pluripotency markers (*OCT3/4*, *NANOG*, *DPPA5A*, *REX1*) and (b) the increase rate of developmental markers (Endoderm: *AFP*, *GATA4* Mesoderm: *ACTA2*, *BRACHYURY*, Ectoderm: *NESTIN*, *PAX6*, Trophoectoderm: *EOMES*) is comparable between WT and Δ9 cells. iPSCs used were derived from WT and Δ9 MEFs of the three established cell lines (b2_1, b2_2 and b3). Data shown are the mean ± SEM from n = 3 independent experiments.

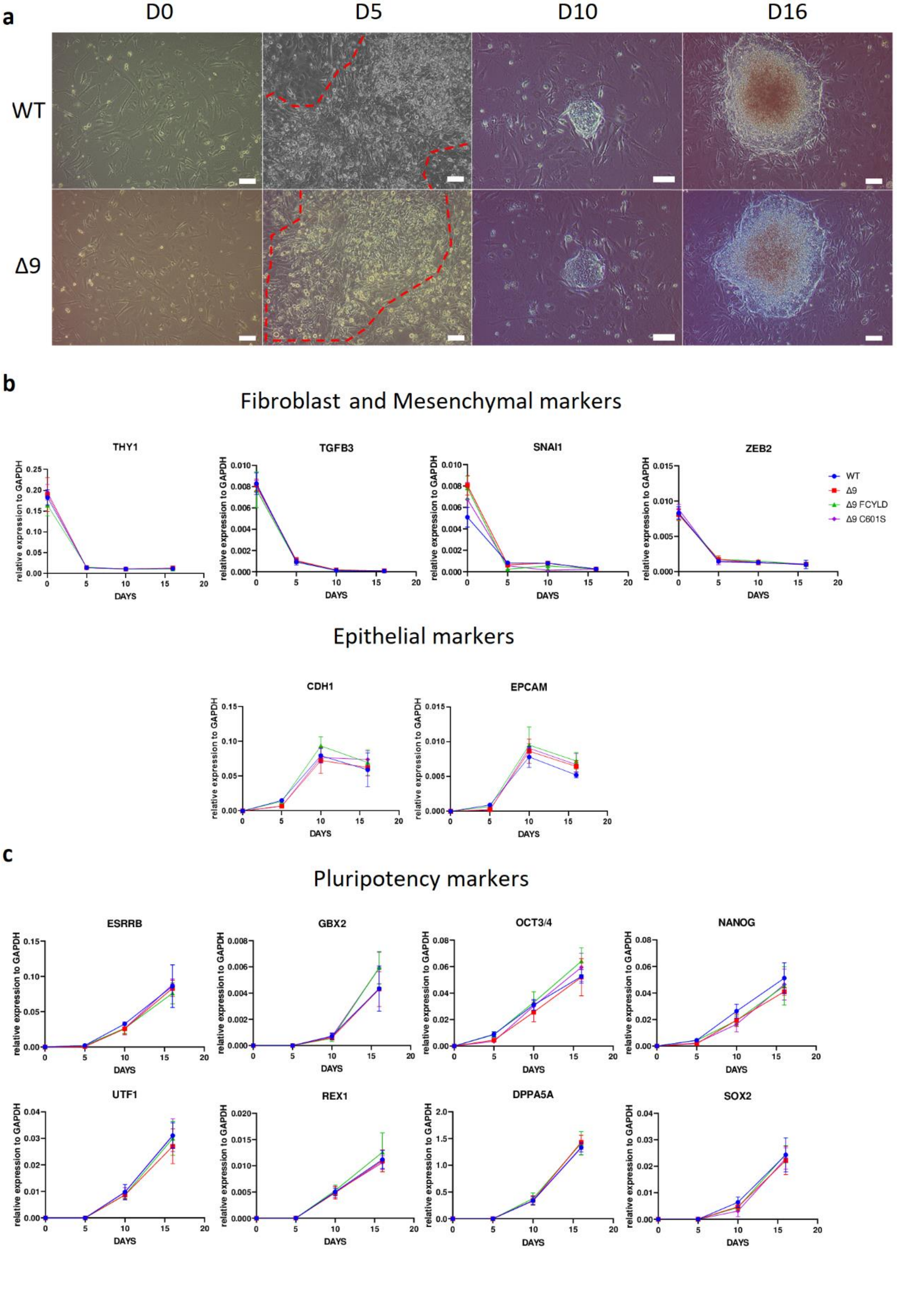

Figure S7. Kinetics of reprogramming. (a) Representative images (scale bar: 100μm) of WT and Δ9 cells harvested during key phases of the reprogramming process (the borders of the D5 early iPSC

colonies are marked with red dashed line). (b-c) Expression of various mesenchymal, fibroblast, epithelial (b) and pluripotency (c) markers in samples representative of the initiation, maturation and stabilization phase of reprogramming. In more detail, on day 10 all pluripotency genes have been sufficiently expressed. Harvested iPSCs were derived from WT and Δ9 reprogrammed MEFs of the three established cell lines (b2_1, b2_2 and b3) and MEFs from the three independent Δ9 FCYLD and Δ9 C601S derivative cell lines. Data shown are the mean ± SEM from *n* = 3 independent experiments

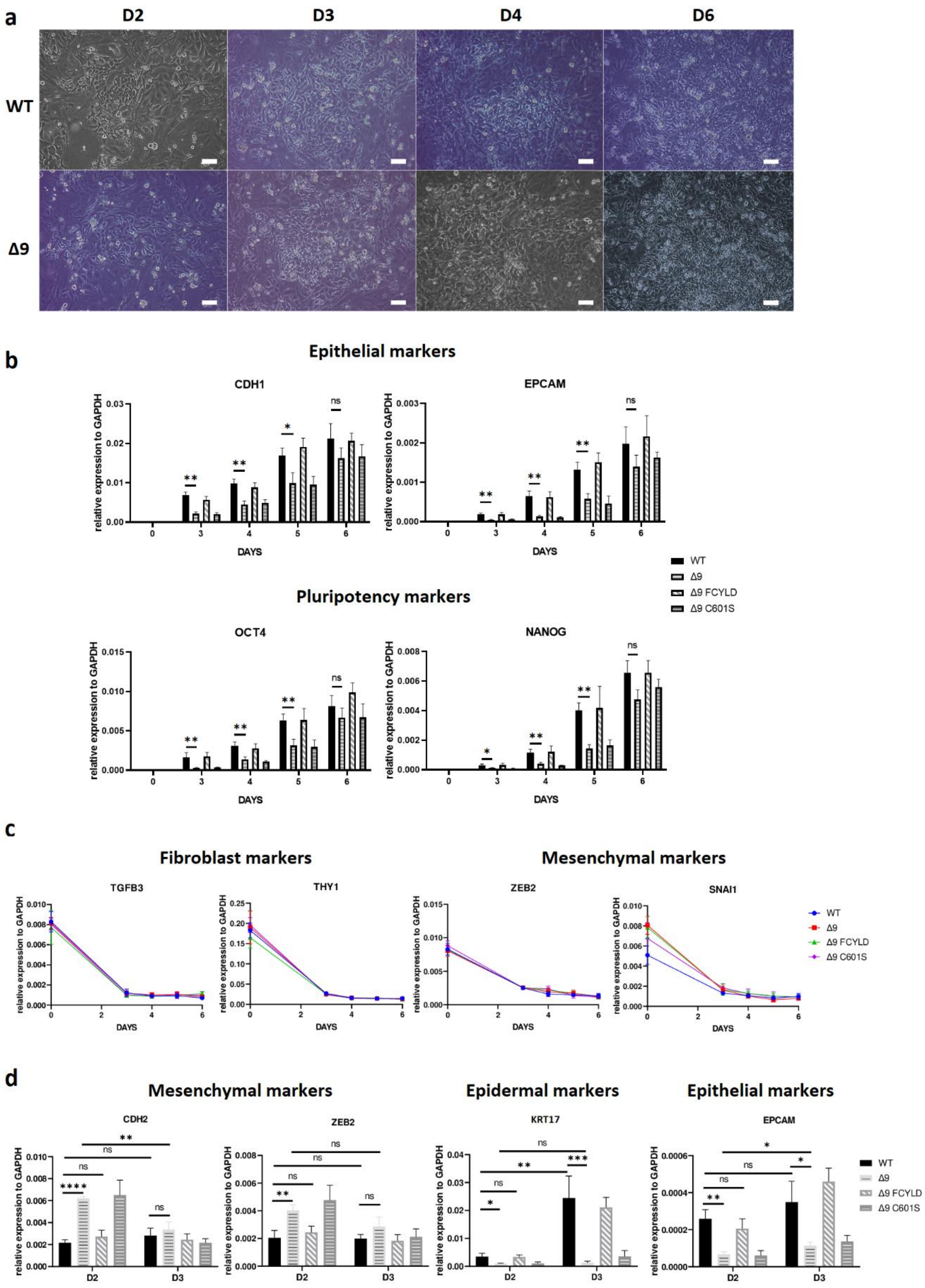

a
D2
D3
D4
D6
WT
Δ9
b
Epithelial markers
CDH1
EPCAM
relative expression to GAPDH
DAYS
ns
WT
Δ9
Δ9 FCYLD
Δ9 C601S
Pluripotency markers
OCT4
NANOG
c
Fibroblast markers
Mesenchymal markers
TGFB3
THY1
ZEB2
SNAI1
d
Mesenchymal markers
Epidermal markers
Epithelial markers
CDH2
ZEB2
KRT17
EPCAM
D2
D3

Figure S8. Kinetics of early reprograming (a) Representative images of early iPSC colony shape during days 2,3 and 4 of reprogramming. (b) The absence of catalytically active CYLD leads to a slower activation of epithelial genes (*CDH1*, *EPCAM*) and endogenous pluripotency (*OCT3/4*, *NANOG*) markers. This effect is more prominent during the first days of early reprogramming. On day 6 there was no observable difference between the four cell types. (c) CYLD DUB deficiency did not affect the downregulation of somatic and mesenchymal markers between days 3-6. (d) Expression levels of mesenchymal (*CDH2*, *ZEB2*), epidermal (*KRT17*) and epithelial (*EPCAM*) markers during D2-D3 transition. Harvested early iPSC colonies are derived from reprogrammed WT and Δ9 MEFs of the three established cell lines (b2_1, b2_2 and b3) and MEFs the three independent Δ9 FCYLD and Δ9 C601S derivative cell lines. Data shown are the mean ± SEM from n = 3 independent experiments, two-tailed Student's t-test (*$p \leq 0.05$, **$p \leq 0.01$, ***$p \leq 0.001$ ****$p \leq 0.0001$. Abbreviation: ns, not significant).

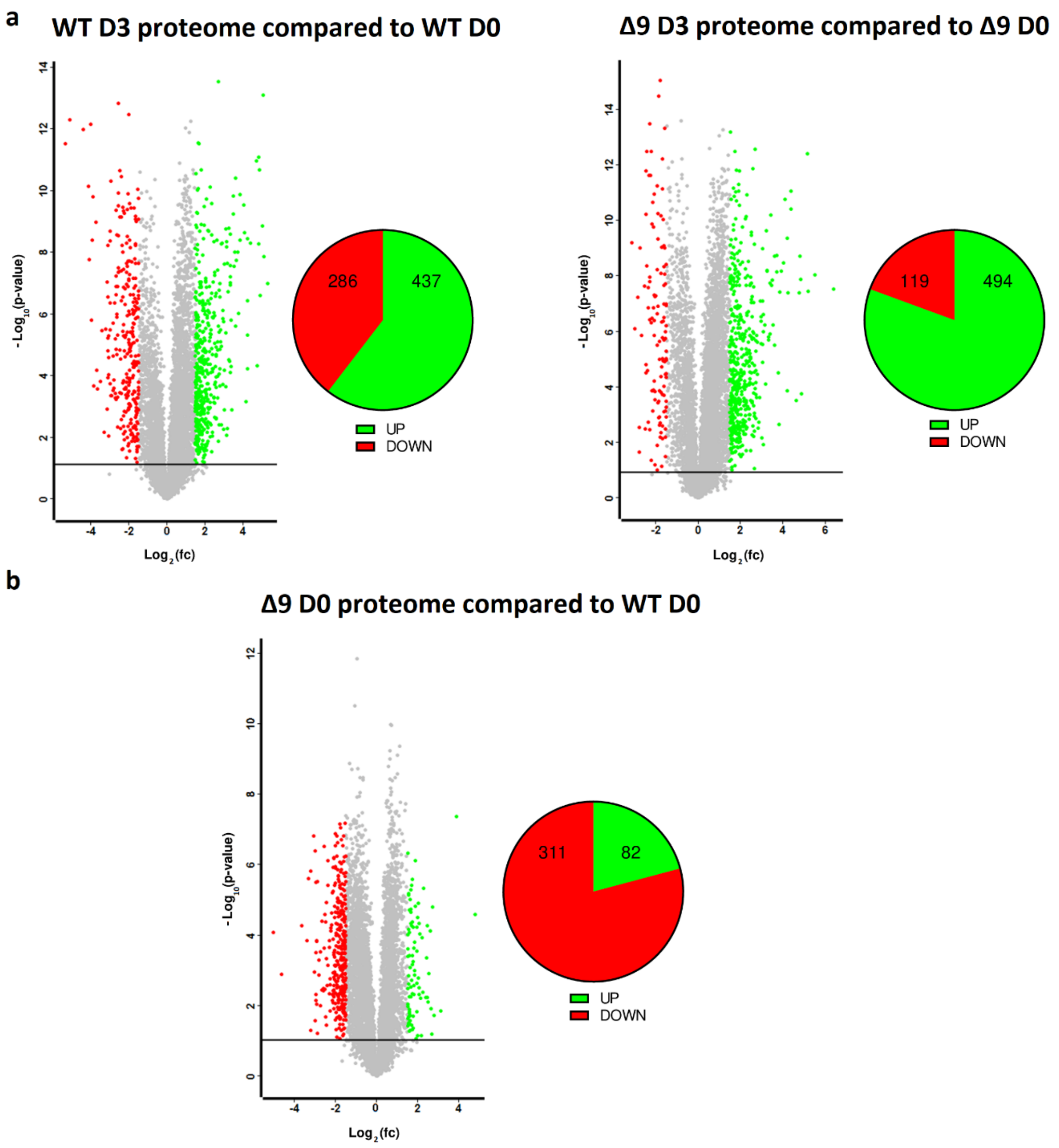

Figure S9. Whole proteome profiling of D0 MEFs and D0-D3 transition. (a) Both WT and Δ9 D0-D3 transitions follow a similar trend, with most proteins being upregulated, although this is more prominent on Δ9 samples (80% of proteins upregulated compared to 60% on WT sampels). On the other hand, total amount of DEPs is slightly higher on WT early iPSCs. (b) Δ9 CYLD affects the proteome of MEFs. More specifically, it has a mostly negative impact on gene expression, since 79% of DEPs are downregulated compared to WT MEFs. In all cases volcano plots were generated with FDR=0.05 and S0=0. A threshold of $|log_2fc \geq 1.5|$ and log_2(p-value) ≥1.5 was applied to determine the statistically significant DEPs. WT and Δ9 D0 MEFs of the three established cell lines (b2_1, b2_2 and b3) were harvested. D3 early iPSCs were derived from these reprogrammed WT and Δ9 MEFs. Each sample was analyzed in at least two technical replicates.

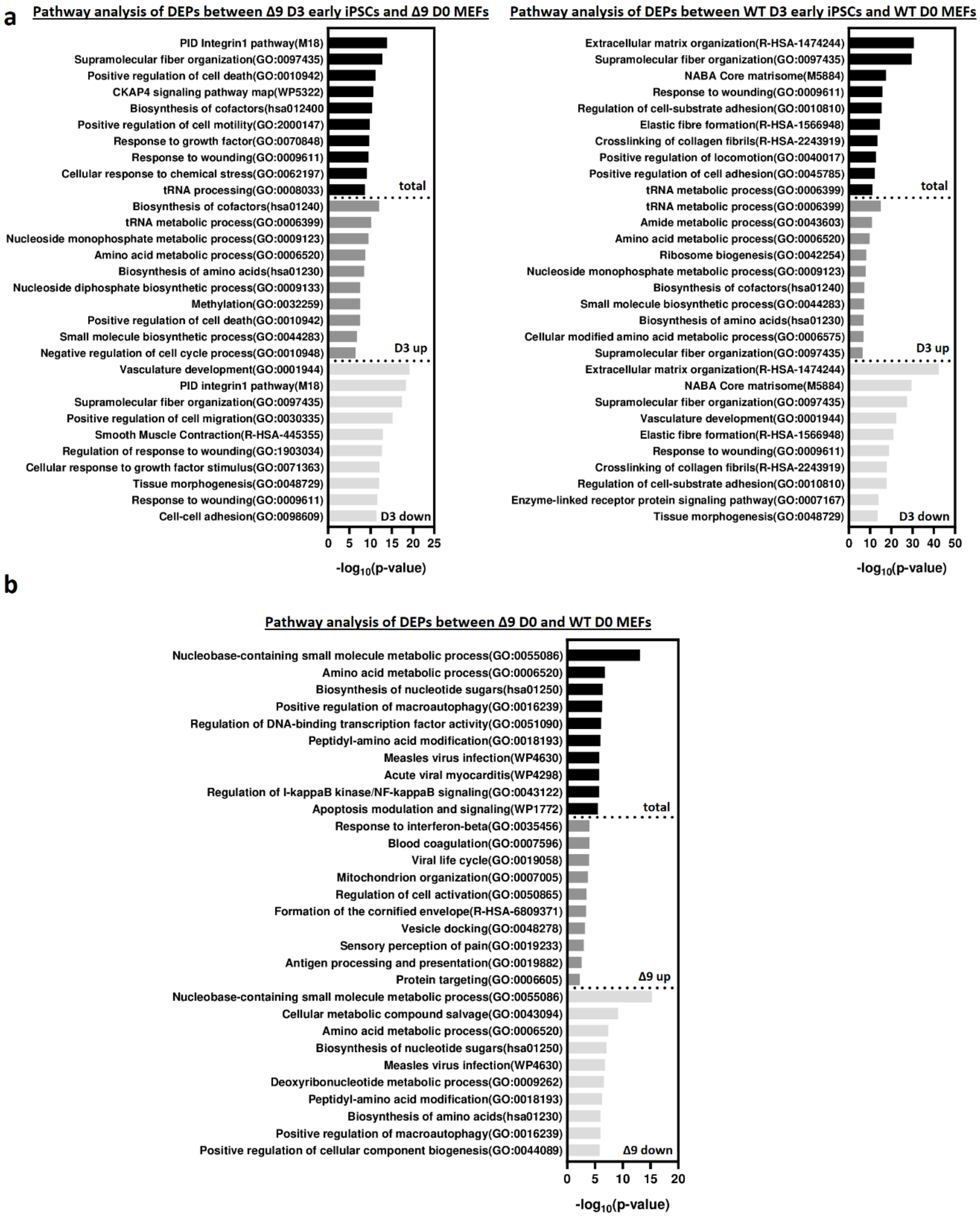

Figure S10. Pathway analysis of D0 MEFs and D0-D3 transitions. (a) In both WT and Δ9 D0-D3 transitions, upregulated DEPs participate in similar processes. However, downregulated DEPs in WT samples present a strong correlation with the extracellular matrix, a process which is completely absent from the pathways associated with Δ9 downregulated DEPs. (b) Most proteins affected by CYLD DUB deficiency in MEFs are associated with various metabolic processes. On the other hand, upregulated DEPs present a weak association with various seemingly unconnected

processes. Interestingly, the combined pathway analysis offer some results that do not appear in either of the aforementioned analyses, such as regulation of macroautophagy, regulation of Nf-kB pathway and modulation of apoptosis.

Table S1. Primer sequences used for qPCR

Gene	***Forward (5’-3’)***	***Reverse (5'-3')***
ACTA2	*GTCCCAGACATCAGGGAGTAA*	*TCGGATACTTCAGCGTCAGGA*
AFP	*CTTCCCTCATCCTCCTGCTAC*	*ACAAACTGGGTAAAGGTGATGG*
BRACHYURY	*AAGCCTTCCTTGATGCCAAA*	*GAGCCTCCAAACTGAGGGTG*
CDH1	*GAAAGCGGCTGATACTGACC*	*CGTACATGTCAGCCGCTTC*
CDH2	*TGTTTGACTATGAAGGCAGTGG*	*TCAGTCATCACCTCCACCAT*
CYLD	*ATTTCCAGGAGTTGTACGCTTC*	*CGTGAAACCTTGACCACGAC*
DPPA5A	*GAAGTCTGGTTCCTTGGCAGGATG*	*ACTCGATACACTGGCCTAGC*
EOMES	*GCGCATGTTTCCTTTCTTGAG*	*GGTCGGCCAGAACCACTTC*
EPCAM	*GCTGGCAACAAGTTGCTCTCTGAA*	*CGTTGCACTGCTTGGCTTTGAAGA*
ESRRB	*GCACCTGGGCTCTAGTTGC*	*TACAGTCCTCGTAGCTCTTGC*
FN1	*GCCCAGAGATAGAGTGACCTG*	*CGCACTTCTCGAAAGTTGCTG*
GAPDH	*CCGCATCTTCTTGTGCAGTG*	*CTGTGGTCATGAGCCCTTCC*
GATA4	*CCCTACCCAGCCTACATGG*	*ACATATCGAGATTGGGGTGTCT*
GBX2	*CAACTTCGACAAAGCCGAGG*	*ACTCGTCTTTCCCTTGCCCT*
KRT17	*ACCATCCGCCAGTTTACCTC*	*CTACCCAGGCCACTAGCTGA*
NANOG	*AGGGTCTGCTACTGAGATGCTCTG*	*CAACCACTGGTTTTTCTGCCACCG*
NESTIN	*CCCTGAAGTCGAGGAGCTG*	*CTGCTGCACCTCTAAGCGA*
OCLN	*CCTCCAATGGCAAAGTGAATGGCA*	*TGTTTCATAGTGGTCAGGGTCCGT*
OCT3/4 ENDOGENOUS	*AGAGTATGAGGCTACAGG*	*AGCTCCAGGTTCTCTTGTCTA*
OCT3/4 TOTAL	*CGGAAGAGAAAGCGAACTAGC*	*ATTGGCGATGTGAGTGATCTG*
PAX6	*GCAGATGCAAAAGTCCAGGTG*	*CAGGTTGCGAAGAACTCTGTTT*
REX1	*ACGAGTGGCAGTTTCTTCTTGGGA*	*TATGACTCACTTCCAGGGGGCACT*
SNAI1	*CACTATGCCGCGCTCTTTC*	*GGTCGTAGGGCTGCTGGAA*
SOX2 ENDOGENOUS	*ACTGCCCCTGTCGCACATGT*	*AGCTGTCGTTTCGCTGCGGA*
SOX2 TOTAL	*GCGGAGTGGAAACTTTTGTCC*	*CGGGAAGCGTGTACTTATCCTT*
TGFB1	*AGGGCTACCATGCCAACTTC*	*CCACGTAGTAGACGATGGGC*
TGFB3	*CCTGGCCCTGCTGAACTTG*	*TTGATGTGGCCGAAGTCCAAC*
THY1	*TGCTCTCAGTCTTGCAGGTG*	*TGGATGGAGTTATCCTTGGTGTT*
UTF1	*TGTCCCGGTGACTACGTCT*	*CCCAGAAGTAGCTCCGTCTCT*

ZEB2	*ATTGCACATCAGACTTTGAGGAA*	*ATAATGGCCGTGTCGCTTCG*
